# Modeling Rett Syndrome With Human Patient-Specific Forebrain Organoids

**DOI:** 10.3389/fcell.2020.610427

**Published:** 2020-12-10

**Authors:** Ana Rita Gomes, Tiago G. Fernandes, Sandra H. Vaz, Teresa P. Silva, Evguenia P. Bekman, Sara Xapelli, Sofia Duarte, Mehrnaz Ghazvini, Joost Gribnau, Alysson R. Muotri, Cleber A. Trujillo, Ana M. Sebastião, Joaquim M. S. Cabral, Maria Margarida Diogo

**Affiliations:** ^1^Department of Bioengineering and iBB-Institute for Bioengineering and Biosciences, Instituto Superior Técnico, Universidade de Lisboa, Lisboa, Portugal; ^2^Instituto de Medicina Molecular João Lobo Antunes, Faculdade de Medicina, Universidade de Lisboa, Lisboa, Portugal; ^3^Instituto de Farmacologia e Neurociências, Faculdade de Medicina, Universidade de Lisboa, Lisboa, Portugal; ^4^The Discoveries Centre for Regenerative and Precision Medicine (Lisbon Campus), Instituto Superior Técnico, Universidade de Lisboa, Lisboa, Portugal; ^5^Department of Pediatric Neurology, Centro Hospitalar Universitário de Lisboa Central, Lisbon, Portugal; ^6^Erasmus MC iPS Facility, Erasmus Medical Center, University Medical Center, Rotterdam, Netherlands; ^7^Department of Developmental Biology, Erasmus Medical Center, University Medical Center, Rotterdam, Netherlands; ^8^Department of Pediatrics, School of Medicine, University of California, San Diego, La Jolla, CA, United States; ^9^Rady Children’s Hospital San Diego, School of Medicine, University of California, San Diego, La Jolla, CA, United States; ^10^Department of Cellular and Molecular Medicine, School of Medicine, University of California, San Diego, La Jolla, CA, United States; ^11^Kavli Institute for Brain and Mind, University of California, San Diego, La Jolla, CA, United States; ^12^Center for Academic Research and Training in Anthropogeny, La Jolla, CA, United States

**Keywords:** human induced pluripotent stem cells, organoids, forebrain, Rett syndrome, disease modeling, neurodevelopmental disorders

## Abstract

Engineering brain organoids from human induced pluripotent stem cells (hiPSCs) is a powerful tool for modeling brain development and neurological disorders. Rett syndrome (RTT), a rare neurodevelopmental disorder, can greatly benefit from this technology, since it affects multiple neuronal subtypes in forebrain sub-regions. We have established dorsal and ventral forebrain organoids from control and RTT patient-specific hiPSCs recapitulating 3D organization and functional network complexity. Our data revealed a premature development of the deep-cortical layer, associated to the formation of TBR1 and CTIP2 neurons, and a lower expression of neural progenitor/proliferative cells in female RTT dorsal organoids. Moreover, calcium imaging and electrophysiology analysis demonstrated functional defects of RTT neurons. Additionally, assembly of RTT dorsal and ventral organoids revealed impairments of interneuron’s migration. Overall, our models provide a better understanding of RTT during early stages of neural development, demonstrating a great potential for personalized diagnosis and drug screening.

## Introduction

Rett syndrome (RTT) is a severe neurological disorder that affects brain development and function in approximately 1 in 10.000 live births, and it is caused by mutations in the gene encoding for methyl-CpG-binding protein 2 (MeCP2). *MeCP2* mutations in females lead to developmental regression after 6–18 months after birth, with a range of neurodevelopmental defects, including loss of speech, acquired movement skills and severe cognitive impairment after an apparently normal development. Less frequent, *MeCP2* mutations in males usually lead to wide spectrum of symptoms, from mild mental retardations to severe congenital encephalopathies and death within the first 2 years of age (Chahrour and Zoghbi, 2007). The range of RTT phenotypes is large and varies according to the mutations, with missense and nonsense mutations accounting for the majority, while small C-terminal deletions and complex rearrangements are less frequent (Chahrour and Zoghbi, 2007). The *MeCP2* gene is localized on the X-chromosome, which causes the disease to be presented in females in a mosaic pattern, with some cells expressing the wild-type *MeCP2* allele and others the mutant (Ananiev et al., 2011).

Rett syndrome has been studied using post-mortem samples of human brain (Colantuoni et al., 2001), as well as transgenic mouse models (Chen et al., 2001). However, the onset of neurological symptoms in mice can be less severe (Veeraragavan et al., 2016) and does not fully recapitulate the phenotypic aspects of human RTT. Recently, RTT was modeled using neural cells differentiated from patient-specific hiPSCs with *MeCP2*-mutant neurons exhibiting impaired maturation, including the presence of fewer synapses, smaller soma size, altered calcium signaling, functional defects in firing activity and excitatory/inhibitory (E/I) imbalance (Marchetto et al., 2010; Ananiev et al., 2011; Fernandes et al., 2015; Tang et al., 2016). However, in the majority of these studies, neural differentiation was performed in 2D adherent monolayer, which provides a simplistic recapitulation of the human cortex development. As a recent innovation in the stem cell field, organoid techniques provide unique platforms to model brain development and neurological disorders (Lancaster et al., 2013). 3D cerebral organoids derived from hiPSCs were recently used for RTT modeling, reveling a decrease in neurite growth, neurite coalescence, and soma size of interneurons (Xiang et al., 2020), in addition to impaired neurogenesis and neural progenitor’s migration deficits (Mellios et al., 2017). However, in cerebral organoids all the regions of the brain are co-generated together, which causes the differentiation process to be poorly controlled and non-region specific (Qian et al., 2016). To overcome these limitations, it is possible to generate independently distinct organoids recapitulating the dorsal and ventral regions of the forebrain (Bagley et al., 2017). *In vivo*, the dorsal forebrain contains the majority of excitatory glutamatergic pyramidal neurons, whereas some evidences in the literature claim that inhibitory *g*-aminobutyric acid (GABA)-producing interneurons are originated in the ventral region, integrating the dorsal cortical circuit upon migration. Ultimately, forebrain multilineage assembloids can be patterned to contain a dorsal–ventral axis and then used to recapitulate human interneuron migration (Bagley et al., 2017; Xiang et al., 2017; Sloan et al., 2018).

In this study, we generated dorsal, ventral and assembled 3D forebrain organoids from RTT patient-derived hiPSCs and their isogenic pair (IC), as well as from several healthy control hiPSCs. These *in vitro* humanized models were used for studying RTT-derived molecular, structural and functional alterations. Intriguingly, our findings suggest that neural progenitors in dorsal organoids derived from RTT female hiPSCs undergo a premature transition into early born neurons, yielding neurons with functional deficits. Our results also propose impairments in the formation of medial ganglionic eminence (MGE) progenitors in ventral organoids, which seems to impact negatively on interneuron’s migration process. Overall, these human *in vitro* 3D models of RTT recapitulate previously reported hallmarks of the disease while providing new clues for the molecular mechanisms involved in this syndrome.

## Materials and Methods

### hiPSC Lines and Maintenance

We used four healthy-control hiPSCs lines and two hiPSCs lines derived from patients with RTT-associated mutations. The healthy-control hiPSC lines were **F002.1A.13** [46, XX cell line derived from a health donor by TCLab (Tecnologias Celulares para Aplicação Médica, Unipessoal, Lda.)], reprogrammed using a standard protocol by Takahashi et al. (2007); **Gibco^®^ iPSC6.2** (46, XY human Episomal cell line, from a healthy donor) and **iPS-DF6-9-9T.B** (46, XY cell line, reprogrammed from foreskin fibroblasts, collected from healthy donors, using defined factors, in the Laboratory of Dr. James Thomson, at University of Wisconsin, and provided by WiCell Bank). All the previous healthy hiPSCs-controls were purchased under MTAs. The RTT cell lines were **EMC25i/WT-R/F7** (46, XX cell line, derived from a healthy donor); **EMC24i/R2 (C6)** [46, XX cell line of a RTT patient with mutation at *MECP2* (R255X), a nonsense mutation on C to T transitions at hypermutable CpG sites within the gene] and **EMC24i/R2 (C5)** (the respective isogenic cell line). The last three cell lines were derived by reprogramming skin fibroblasts ensuring data protection issues. Human skin punch biopsy samples (3 mm) were collected using a standard technique. The protocol was established with the Paediatric Surgery Department at Centro Hospitalar de Lisboa Norte (CHLN) to collect skin samples during minor surgeries. Biopsy tissue was expanded at Instituto de Medicina Molecular, Lisboa. Hospital Sant Joan de Déu (HSJD) and CHLN Ethic Committees approved the study and written informed consent was obtained from patient’s legal guardians. Then, the reprogramming of fibroblasts was performed at the iPSC Facility, Erasmus Medical Center, Rotterdam, using engineered color-coded lentiviral vectors (Varga et al., 2016). Moreover, a **RTT male cell line was also used, Rett Male: Q83X** (46, XY cell line, derived from fibroblasts of a RTT male patient with a mutation at the amino acid residue 83 from glutamine to a premature stop codon, resulting in truncation and degradation of the MeCP2 protein). This cell line was donated by Alysson Muotri Lab at UCSD, United States, under an MTA. These cells were reprogrammed with retroviral reprogramming vectors (Sox2, Oct4, c-Myc, and Klf4) (Takahashi and Yamanaka, 2006), and provided by University of California, San Diego Campus and approved by the institutional review boards at Salk Institute for Biological Studies and University of California, San Diego, as well as Pennsylvania State University (Marchetto et al., 2010).

All the hiPSCs lines were cultured on Matrigel^TM^ (Corning)-coated plates with either Essential 8^TM^ Medium (Thermofisher Scientific) or mTeSR^TM^1 Plus Medium (StemCell Technologies). Medium was changed according to the respective procedures. Cells were passaged every 3–4 days (at approximately 85% of cell confluence) using 0.5 mM EDTA dissociation buffer (Thermofisher Scientific). Before starting the differentiation process, two to three passages were performed.

### 3D Induction of Dorsal and Ventral Forebrain Organoids

Before starting the ventral and dorsal neural induction protocols, the hiPSC were incubated with ROCK inhibitor (ROCKi, Y-27632, 10 μM, StemCell Technologies) for 30 min at 37°C and then treated with accutase (Sigma) for 5 min at 37°C. After dissociation, cells were seeded on microwell plates (AggreWell^TM^800, StemCell Technologies) according to the manufacturer’s instructions. The cell density used was 1.5 × 10^6^ cells/well in 1.5 mL/well of E8^TM^ or mTeSR^TM^1 Plus supplemented with 10 μM ROCKi. The entire expansion medium was changed after 24 h, without ROCKi supplementation. Usually after 2–3 days, when the aggregates attained a diameter of 250–300 μm, the medium was half-changed to induction medium, and this day was defined as day 0. The neural induction medium used was N2B27, composed by 50% of DMEM/F12/N2 (DMEM-F12, (Thermofisher Scientific) supplemented with 1% (v/v) N2 (Thermofisher Scientific), 1.6 g/L Glucose (Sigma), 1% (v/v) PenStrep, and 20 μg/mL Insulin (Sigma) and 50% of Neurobasal/B27 [Neurobasal medium (Thermofisher Scientific) supplemented with 2% (v/v) B27(-Vitamin A)-supplement (Thermofisher Scientific), 2 mM L-glutamine (Thermofisher Scientific) and 1% (v/v) PenStrep]. For the dorsal forebrain patterning, the previously described medium was supplemented with 2 μM Dorsomorphine (Sigma) and 2 μM A83-01 (Tocris) until day 5, with half-medium changed at days 0, 3 and 5. For the ventral patterned aggregates, the medium was supplemented with 10 μM SB-431542 (SB) (Sigma) and 100 nM LDN-193189 (LDN) (Stemgent). At day 5, cell aggregates (around 300 aggregates per each well of the microwell plate used) were collected from the microwell plates and seeded in an Ultra-Low attachment 6-well plate (Corning). At day 7, half of the medium was changed for both dorsal and ventral patterning. In dorsal patterning cultures, the medium was supplemented with 1 μM CHIR99021 (Stemgent), 10 μM SB-431542 (Sigma) and 10 μg/ml Heparin (Sigma) and ventral patterning culture medium was supplemented with 2.5 μM IWP2 (Stemgent), 100 nM SAG (Millipore) and 10 μg/ml Heparin (Sigma). When required, on day 13, the assembly of one dorsal with one ventral organoid was performed inside a 96-TC Plate, Suspension, C (Sarstedt) and after 24 h the fused organoid was transferred into an Ultra-Low attachment 24-well plate.

### Maintenance and Maturation of Dorsal, Ventral and Fused Organoids

On day 13, the medium was half-changed to N2B27 (+Vitamin A) without any small molecule supplementation and was maintained in the presence of this medium until day 41. This medium was replaced every 2/3 days. On day 41 of differentiation, the organoids were cultured in BrainPhys^TM^ Neuronal Medium (StemCell Technologies), supplemented with NeuroCult^TM^ SM1 Neuronal Supplement (StemCell Technologies), N2 Supplement-A (StemCell Technologies), Recombinant Human Brain Derived Neurotrophic Factor (BDNF, PeproTech, 20 ng/mL), Recombinant Human Glial-Derived Neurotrophic Factor (GDNF, PeproTech, 20 ng/mL), dibutyryl cAMP (1 mM, Sigma), and ascorbic acid (200 nM, Sigma). One third of the total volume was replaced every 2–3 days.

### Tissue Preparation and Immunostaining

Organoids replated in coverslips were fixed in 4% (w/v) PFA (Sigma) for 20 min at 4°C, followed by washing in phosphate buffered saline (PBS, 0.1M). Whole 3D organoids were fixed in 4% PFA for 30 min at 4°C, with agitation, followed by washing in PBS 0.1M and overnight incubation in 15% (w/v) sucrose at 4°C. Then, they were embedded in 7.5% gelatin/15% sucrose and isopenthane (Sigma) was subsequently used for freezing at −80°C. A cryostat-microtome (Leica CM3050S, Leica Microsystems) was used to prepare organoid sections with approximately 12 μm thickness and collected on Superfrost^TM^ Microscope Slides (Thermo Scientific) (stored at −20°C). Organoid sections were de-gelatinized in PBS at 37°C for 45 min and then analyzed by immunohistochemistry. Organoid sections and cells plated on coverslips were then incubated in 0.1 M Glycine (Millipore) for 10 min at room temperature (RT), permeabilized with 0.1% Triton X-100 (Sigma) for 10 min at RT, and blocked with 10% fetal bovine serum (FBS, Thermofisher Scientific) in TBST [20 mM Tris-HCl pH 8.0, 150 mM NaCl, 0.05% (v/v) Tween-20, Sigma] for 1 h at RT. Then, primary antibodies were diluted in blocking solution and incubated overnight at 4°C. After three washing steps with TBST, secondary antibodies were incubated for 45 min at RT. For phalloidin staining, cells were incubated with Alexa Fluor^®^ 488 Phalloidin during 30 min (1:50 in PBS, Thermofisher Scientific). For GFP staining, cryosections were incubated with anti-GFP polyclonal IgG, Alexa Fluor^®^–488. Nuclear counterstaining was performed using 4′,6-diamidino-2-phenylindole (DAPI, 1.5 μg/mL; Sigma). After drying, sections and coverslips were mounted in Mowiol (Sigma). Fluorescence images were acquired using Zeiss LSM 710 Confocal Laser Point-Scanning Microscopes and images were processed in ZEN 2.3 blue edition software (Zeiss).

### Flow Cytometry

Samples were collected with accutase (Sigma) for 5 min at 37°C and then stored in 2% w/v PFA (Sigma). Samples were centrifuged at 1000 rpm for 5 min and washed twice with PBS. Samples for KI67 analyzes were also collected with accutase, and then fixed drop by drop with 70% (v/v) ethanol (−20°C) with vortex. Samples were stored at −20°C, being then centrifuged at 1000 rpm for 10 min and washed twice with PBS. The Eppendorf tubes were coated with 1% v/v bovine serum albumin (BSA; Thermofisher Scientific) solution in PBS for 15 min. For intracellular staining, cell were resuspended in 3% (v/v) normal goat serum (NGS, Sigma), at approximately 1 × 10^6^ cells per condition. The cell suspension was centrifuged again at 1000 rpm for 3 min. The cell membrane was then permeabilized using a solution 1:1 of 3% (v/v) NGS and 1% (v/v) saponin (Sigma) for 15 min, at RT. After washing three times with 1% (v/v) NGS, cells were resuspended in primary antibody solution (in 3% NGS) and incubated for 1 h at room temperature. Cells were then washed three times with 1% NGS, and incubated for 30 min in the dark with the secondary antibody (in 3% NGS) and 300 μL were transferred to a FACS (flow cytometry) tube. For surface staining, cells were resuspended in primary antibody diluted in 10% (v/v) FBS in PBS, at approximately 0.5 × 10^6^ cells per condition, and incubated for 30 min at RT. Finally, cells were washed with PBS and resuspended in 10% (v/v) FBS in PBS and incubated with secondary antibodies for 15 min, at room temperature. After another washing step, cells were resuspended in PBS and 300 μL were transferred to a flow cytometry tube. Flow cytometry was performed using a FACSCalibur^TM^ flow cytometer (by Becton Dickinson) and the data acquisition was performed with the Cell Quest software (Becton Dickinson). A minimum of 10,000 events were analyzed for each sample. The data analysis was performed using Flowing Software 2.0. As negative controls, unstained samples and samples stained with only the secondary antibody were used, without showing relevant differences between them. The gate was selected to contain less than 1% of false positives (i.e., 1% of the negative control samples). Primary antibodies used for flow cytometry were SSEA-4-PE (Miltenyi Biotec, 1:10) for surface staining and OCT4 (Invitrogen, 1:300) and KI67 (BD, 1:100) for intracellular staining. Secondary antibodies used were goat anti-mouse IgG Alexa Fluor – 488 (Invitrogen, 1:300) and anti-mouse IgG-PE (1:300, Miltenyi Biotec).

### Quantitative Real Time (qRT)-PCR

At different time points of differentiation, RNA samples were extracted by using High Pure RNA Isolation Kit (Roche) and converted into complementary cDNA with Transcriptor High Fidelity cDNA Synthesis Kit (Roche). Real-time quantitative PCR (qPCR) was performed using the StepOne^TM^ or the ViiA^TM^ 7 RT-PCR Systems (Applied BioSystems). Taqman^®^ Gene Expression Assays (20X, Applied Biosystems) were selected for *NANOG*, *PAX6*, *TBR1, DLX2, NKX2.1, LHX6, FOXG1* and *GAPDH*. *DLL1*, *HES5*, *PARVALBUMIN (PV*), *VGLUT1* and *GAPDH* analysis were performed using the SYBR Green Master Mix (Nzytech). The results were analyzed with the StepOne^TM^ or the QuantStudio^TM^ RT-PCR Software. All PCR reactions were done in duplicate or triplicate and then normalized to the housekeeping gene *GAPDH*. The fold change was calculated using the 2^Δ*Ct*^ method and in some graphical results it was calculated relatively to the control condition levels obtained. The representative heatmaps were generated using the web tool Clustvis (Metsalu and Vilo, 2015).

### RNA *in situ* Hybridization

The probes were generated by using a template cDNA, amplified by PCR, with the reverse primers containing the T7 site: *DLL1-*fw (TGTGCCTCAAGCACTACCAG) rv(+T7) (TA ATACGACTCACTATAGGGATGCTGCTCATCACATCCAG);

*HES5*-fw (ACGCAGATGAAGCTGCTGTA) rv(+T7) (TA ATACGACTCACTATAGGGGGCCCTGAAGAAAGTCCTCT). The PCR products were gel-purified using the NZYGelpure kit (NZYTech) and used for reverse transcription with T7 polymerase (Agilent Technologies), DIG-dNTPs (Sigma), and RNase inhibitor. The RNA was precipitated with EtOH and NaOAC_3_ at 20°C overnight, centrifuged 30 min at 13.000 *g*, and then washed with 70% EtOH, dried, and resuspended in nuclease-free H_2_O with 10mM of EDTA (stored at 20°C until use).

The organoids were fixed in 4% PFA (w/v) during 1 h at 4°C. Fresh cryosections (12 μm) were incubated with 10 nM FISH probes, overnight, at 65°C in hybridization buffer, containing 1x salts, 10% dextran sulfate, 1 mg/mL rRNA, 1x Denhardt’s and 50% of deionized formamide (Sigma). After hybridization, the cells were washed twice, for 30 min at 65°C, using a washing buffer [10% formamide (Sigma) in 2 × SSC] and then washed at RT with TBST. The slices were then blocked using TBST with 2% (v/v) blocking reagent (Roche) and 20% (v/v) heat inactivated sheep serum (Sigma) for 1h at RT. The slices were then stained with antibody anti-DIG (Abcam) diluted into TBST, 2% (v/v) blocking reagent, 1% sheep serum and left to incubate overnight, at 4°C. After incubation, slices were washed four times with TBST. Afterward, organoid slices were incubated twice during 10 min in 0.4M NaCl, Tris 0.1M (pH8). The incubation was then performed with Fast-red tablets substrate (Sigma) diluted in 0.4M NaCl, Tris 0.1M (pH8) for 1–3 h in the dark until the visible spots appear, and then the reaction was stopped with PTW [PBS + 0.1% (v/v) Tween-20]. Immunofluorescence staining was then performed. Images were acquired using Zeiss LSM 710 Confocal Laser Point-Scanning Microscopes and processed in ZEN 2.3 blue edition software (Zeiss).

### Single Cell Calcium Imaging (SCCI)

The organoids used in calcium imaging recordings were first dissociated gently with accutase, 4–5 days before the recordings, and replated into matrigel-coated 8-well chambers Lab-TekTM Chamber Slide (ThermoFisher Scientific). Before the experiments, cells were loaded with 5 μM Fura-2 AM (Invitrogen) in Krebs solution (132 mM NaCl, 4 mM KCl, 1.4 mM MgCl2, 2.5 mM CaCl_2_, 6 mM glucose, 10 mM HEPES, pH 7.4) for 45 min at 37°C in an incubator with 5% CO_2_ and 95% atmospheric air (Silva et al., 2020). Cells were washed in Krebs solution and then mounted on an inverted microscope with epifluorescence optics (Axiovert 135TV, Zeiss). Cells were continuously perfused with Krebs solution and stimulated by applying high-potassium Krebs solution (containing 50 mM KCl, isosmotic substitution with NaCl) or 100 μM histamine. The time course of SCCI experiments was the following: 300 s baseline, stimulation with KCl from 300 to 420 s, KCl washout from 420 to 600 s, stimulation with histamine from 600 to 720 s and histamine washout from 720 to 900 s recordings. Following that, Histamine/KCl ratios were calculated using the corresponding peak values given by normalized ratios of fluorescence at 340/380 nm from image pairs acquired every 200 ms by exciting the cells at 340 and 380 nm. Mature neurons typically depict ratios below 0.8 (Agasse et al., 2008; Bernardino et al., 2013). Excitation wavelengths were changed through a high-speed switcher (Lambda DG4, Sutter Instrument, Novato, CA, United States). The emission fluorescence was recorded at 510 nm by a cooled CCD camera (Photometrics CoolSNAP fx). Images were processed and analyzed using the software MetaFluor (Universal Imaging, West Chester, PA, United States). Regions of interest were defined randomly and manually over the cell profile.

### Patch-Clamp Recordings

Organoids were first dissociated gently with accutase, 4–5 days before the recordings, and replated into glass cover-slips previously coated with matrigel. Whole-cell patch-clamp recordings were visualized with an upright microscope (Zeiss Axioskop 2FS) equipped with differential interference contrast optics using a Zeiss AxioCam MRm camera and a x40 IR-Achroplan objective. During recordings, cells were continuously superfused with artificial cerebrospinal fluid (aCSF) containing 124 mM NaCl, 3 mM KCl, 1.2 mM NaH2PO_4_, 25 mM NaHCO_3_, 2 mM CaCl_2_, 1 mM MgSO_4_, and 10 mM glucose, which was continuously gassed with 95%O_2_/5%CO_2_. Recordings were performed at room temperature in current-clamp or voltage-clamp mode [holding potential (Vh) = −70 mV] with an Axopatch 200B (Axon Instruments) amplifier (Dias et al., 2014). The step-and-hold stimulation protocol included 11 steps of 500 ms long depolarization pulses. The first injection current was −25 pA and the subsequent ones increased progressively until 225 pA.

Action potential activity was recorded using patch pipettes (4–9 MΩ resistance) pulled from borosilicate glass capillaries (1.5 mm outer diameter, 0.86 mm inner diameter, Harvard Apparatus, Holliston, MA, United States) with a PC-10 Puller (Narishige Group, London, United Kingdom) filled with an internal solution containing 125 mM K-gluconate, 11 mM KCl, 0.1 mM CaCl2, 2 mM MgCl2, 1 mM EGTA, 10 mM HEPES, 2 mM MgATP, 0.3 mM NaGTP, and 10 mM phosphocreatine, pH 7.3, adjusted with 1 M NaOH, 280–290 mOsm. Acquired signals were filtered using an in-built, 2-kHz, three-pole Bessel filter, and data were digitized at 5 kHz under control of the pCLAMP 11 software programs (Molecular Devices, San José, CA, United States). The junction potential was not compensated for, and offset potentials were nulled before gigaseal formation. The resting membrane potential was measured immediately after establishing whole cell configuration and the junction potential of the electrode was considered (−12 mV). Firing patterns of neurons were determined in current-clamp mode immediately after achieving whole-cell configuration by a series of hyperpolarizing and depolarizing steps of current injection (1 s). In the voltage-clamp mode, spontaneous miniature postsynaptic currents were recorded in a CSF solution during 5 min. Analysis of the amplitude and half-peak within were performed off line using the Clampfit 11 software. AP velocity (dV/dt) in a train of AP firing near threshold was represented from the first derivate of the first AP generated.

### Lentiviral Transfection for Derivation of GFP^+^ hiPSCs Lines

HEK 293T cells were used for lentiviral production, followed by concentration by ultra-centrifugation. Briefly, a second generation packaging system composed of three plasmids (transfer vector with expression construct LV-GFP, the packaging plasmid psPAX2, and the envelope protein expression plasmid pMD2.G – pMD2.G, a gift from Didier Trono (Addgene plasmid # 12259^1^; RRID: Addgene_12259), was mixed in a ratio of 2:*1*:1 in DMEM (Thermofisher Scientific) and Fugene6 transfection reagent (Roche) and 293T cells were transfected during 4 h to overnight. The supernatant from the transfected plate was collected every 24 h in serum-free conditions and concentrated by ultracentrifugation (Beckman XL-90) at 90.000 rpm for 2 h at 4°C. The concentrated viral solution was passed through a 0.45 μm low-protein binding filter and aliquots were used to transfect the hiPSCs lines. The cell lines F002.1A.13, Gibco^®^ iPSC6.2 and EMC24i/R2 (C6) were successfully transfected, by first incubating the cells in a 24-well plate during 30 min at 37°C with E8 medium, 10 μM ROCKi and 5 μg/mL of Polybreme (Sigma). Then, LV-GFP previously dissolved in E8 was added dropwise and the cells were incubated during 1 h30 min. Culture medium was then changed daily. After cell growth and expansion, FACS sorting of GFP^+^ cells was performed using BD FACSAria^TM^ III (BD Biosciences-US) in order to obtain pure populations.

### Statistical Analysis

The data were expressed as mean of ± standard error of mean (SEM) from at least three independent n experimental replicates/differentiations, as n number of organoids analyzed or n neurons (SCCI and patch-clamp recordings). Mean differences between control cell lines and RTT lines were evaluated by two-tailed unpaired and non-parametric *t*-test. Statistical processing was performed using Microsoft Excel and GraphPad Prism Software. Statistical significance level was set for (^∗^) *p*-values < 0.05; (^∗∗^) *p*-values < 0.01; (^∗∗∗^) *p*-values < 0.001. The statistical details of experiments are also present in the figure legends.

### Data Availability

The authors declare that all raw data presented in all the figures of the manuscripts that support the findings of this study are available from the corresponding author upon request.

## Results

### MeCP2:R255X Dorsal Forebrain Organoids Reveal Premature Formation of Cortical Plate Neurons

Glutamatergic pyramidal neurons arise mainly from progenitors in the dorsal region of the forebrain (the pallium), specifically from radial glial cells (RGs), found in the VZ, and from outer radial glial cells (oRGs) and intermediate progenitors (IPs), found in the subventricular zone (SVZ) (Lui et al., 2011). For modeling RTT-derived alterations in the process of neurogenesis in the dorsal forebrain, we engineered organoids resembling this brain region. Dorsal organoids were generated from four distinct female and male healthy control hiPSCs and two patient-specific RTT hiPSC lines (female MeCP2:R255X and male MeCP2:Q83X). It is known that, in females, random inactivation of the X-chromosome occurs during development (Balachandar et al., 2016). Therefore, as *MeCP2* gene is located in the long arm of the X chromosome, RTT female patients are characterized by the presence of somatic mosaicism. Since female hiPSCs retain an inactive X-chromosome in a non-random pattern (Tchieu et al., 2010), it was possible to obtain an isogenic control (IC-MeCP2:R255X) from a distinct clone isolated upon reprogramming of the mosaic female cell line (Supplementary Figure 1.1A). Upon differentiation, IC cells exhibited only the non-mutated *MeCP2* active X-chromosome. The presence/absence of the mutations was confirmed by Sanger sequencing of gDNA and cDNA (Supplementary Figure 1.1B). The pluripotent phenotype of the IC and female and male RTT hiPSC lines was confirmed by flow cytometry (Supplementary Figure 1.1C).

**FIGURE 1:**
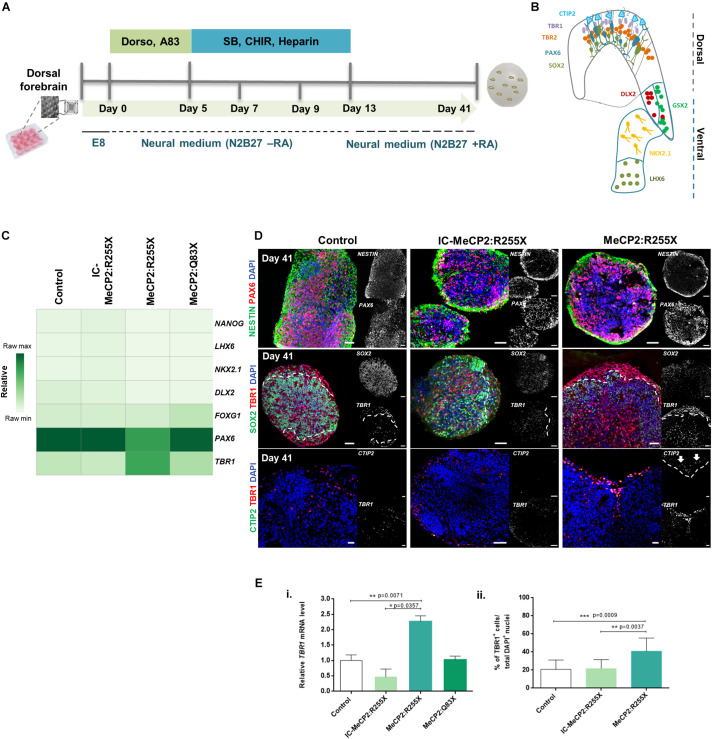
Dorsal forebrain organoids derived from RTT-specific hiPSCs reveal a premature formation of the cortical plate. **(A)** Schematic overview of the protocol for dorsal forebrain organoid development until day 41 of differentiation. **(B)** Schematic overview of the two forebrain regions of the mammalian cortex presenting the characteristic markers/populations for dorsal and ventral regions. **(C)** Transcriptional profile of dorsal forebrain organoids at day 41 of differentiation. Quantitative RT-PCR analysis of the pluripotency marker *NANOG*, the forebrain ventral characteristic markers *LHX6, NKX2.1* and *DLX2*, the generic forebrain marker *FOXG1* and the dorsal characteristic markers *PAX6* and *TBR1*. All values were normalized and relative to *GAPDH*. Raw minimum (min) and raw maximum (max) values were taken as a reference for heatmap representation. (*n* = independent experiments. Control *n* = 10; IC *n* = 5; MeCP2:R255X *n* = 4; MeCP2:Q83X *n* = 4). **(D)** Immunofluorescence characterization of dorsal organoids at day 41. Representative images of dorsal organoid sections were stained against PAX6, NESTIN and SOX2 (neural progenitor markers), TBR1 (marker for new-born neurons of the deep cortical layer VI), and CTIP2 (marker for neurons of deep cortical layer V). Scale bars, 50 μm. **(E) (i)** Relative mRNA levels of *TBR1* on dorsal organoids at day 41 of differentiation already represented on the heatmap **(C)**. mRNA levels relative to *GAPDH* and normalized to control condition. *n* = independent experiments; Control *n* = 10; WT-MeCP2:R255X *n* = 5; MeCP2:R255X *n* = 4; MeCP2:Q83X *n* = 4. For all graphics depicted, Student’s *t*-test (two-tailed) statistics, was applied *p < 0.05, **p < 0.01, ***p < 0.001; error bars represent SEM. **(ii)** Immunocytochemistry quantification of the percentage (%) of cells expressing TBR1 normalized to the total number of cells stained with DAPI. TBR1: Control *n* = 3 (12 organoids); IC-MeCP2:R255X *n* = 3 (8 organoids); MeCP2:R255X *n* = 3 (10 organoids). *n* = independent experiments. For all graphics depicted, Student’s *t*-test (two-tailed) statistics, was applied *p < 0.05, **p < 0.01, ***p < 0.001; error bars represent SEM.

The dorsal forebrain organoids were generated according to the dual-SMAD inhibition protocol and cultured until day 41 by adapting previously described methodologies (Qian et al., 2016) (Figure 1A). As an important modification, hiPSCs were cultured in Aggrewell^TM^ 800 plates during the 5 days of neural induction (Miranda et al., 2015, 2016, 2018). This strategy allowed the generation of size-controlled aggregates, with a narrow size-distribution (diameter 250–300 μm) (Supplementary Figure 1.1D), allowing a more homogeneous feeding of soluble factors across the aggregate. At day 13 of dorsal induction, histological analysis of organoid cryosections, revealed the presence of cells expressing the forebrain specific progenitor marker OTX1/2 in all dorsal organoids (see Supplementary Figure 1.1E). Moreover, the formation of SOX2^+^/NCAD^+^ neural rosette-like structures, resembling the neural tube, was also observed (see Supplementary Figure 1.1F). All dorsal organoids revealed the presence of SOX2+ progenitor cells, resembling the proliferative regions of the human VZ. In addition, the rosette structures exhibited the cadherins junction marker N-CAD, located at the luminal side (see Supplementary Figure 1.1F).

To further confirm the dorsal identity of these organoids, we examined the mRNA expression levels of dorsal, ventral and pluripotency markers at day 41 of differentiation by qRT-PCR (Figures 1B,C). Expression of *FOXG1* was observed in all organoids, confirming their forebrain identity. The ventral layer markers, *DLX2, NKX2.1* and *LHX6*, and the pluripotency marker *NANOG*, were almost undetectable. It was also detected a high expression of the neural progenitor dorsal-cortical marker *PAX6*, and of *TBR1*, a marker typically expressed in neurons of deep cortical layer VI (Hevner et al., 2001). No significant differences were found when comparing the mRNA levels of the male-RTT organoids with the ones of the control organoids. For this reason, we focused our subsequent analyzes on the RTT female cell line, the respective IC and the healthy-controls. Interestingly, female RTT dorsal organoids exhibited a statistically significant higher expression of *TBR1* (see Figure 1Ei) when compared with the IC and healthy-control dorsal organoids, and a statistically significant lower expression of *PAX6* (see Supplementary Figure 1.2A) when compared to the healthy-control organoids. A specific oRG cells marker, HOPX, was used to verify the presence of a prominent oRGC-like population (Qian et al., 2016). On Supplementary Figure 1.2B, by immunocytochemistry, it was possible to observe the presence of that progenitor layer, which is characteristic of human cortical development. However, a narrower expression pattern was detected for the Rett female cell line organoids in comparison to control organoids. Moreover, immunocytochemistry analysis of organoid sections showed that control organoids developed a well-defined TBR2^+^ IP cell region, confirming the presence of a SVZ layer, an important hallmark of human cerebral cortex development. Conversely, female RTT dorsal organoids lacked SVZ formation, as observed by the absence of TBR2^+^/SOX2^+^ cells (Supplementary Figure 1.2C). In line with this observation, quantification of the number of TBR2^+^ cell nuclei confirmed the presence of these progenitor cells in healthy-control (16 ± 4%) and IC (18 ± 2%) organoids and the almost complete absence of these cells in female RTT organoids (Supplementary Figure 1.2D). We further detected that TBR1 was mainly expressed superficially to the progenitor SOX2^+^ VZ-like layer (Figure 1D). Importantly, TBR1 protein was expressed in a higher percentage of cells in the female RTT organoids (41 ± 5%), when compared with the healthy-control (21 ± 3%) and with the IC (22 ± 4%) organoids (Figure 1Eii). Furthermore, RTT female organoids exhibited the presence of CTIP2^+^ cells, indicating the specification of a deep cortical plate layer, which at this time-point is not yet observed in healthy-control and IC organoids (Figure 1D).

Overall, dorsal forebrain organoids derived from the RTT mutant female hiPSCs revealed a higher expression and premature formation of newborn deep-layer cortical neurons, in parallel with a depletion of IP cells and a decrease in the number of oRG cells. These results suggest that for the hiPSC lines exhibiting the MeCP2:R255X mutation, molecular and structural alterations start to occur early in development, during the process of formation of cortical layers in dorsal forebrain consistent with a premature neuronal differentiation process.

### Premature Differentiation in MeCP2:R255X Dorsal Organoids

**FIGURE 2:**
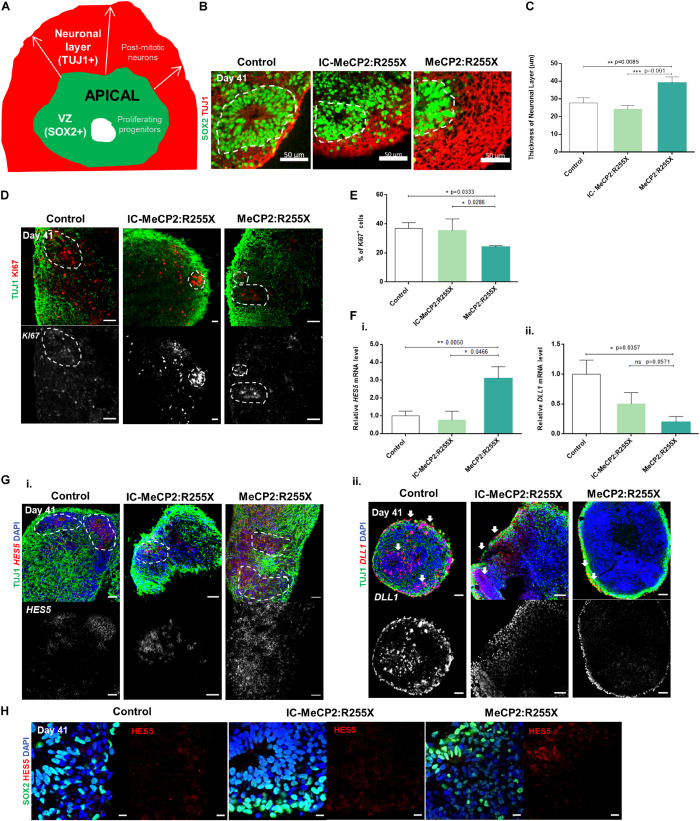
Altered proliferation pattern and dynamics of expression of Notch signaling operands *HES5* and *DLL1* during differentiation of RTT female patient-derived dorsal organoids. **(A)** Schematic representation of SOX2^+^ ventricular zone (VZ) and TUJ1^+^ neuronal layer measurement in cortical structures. For each cortical structure, three measurements were taken for the post-mitotic neuronal zone. **(B)** Representative images of the VZ delineated by white dashes and the neuronal layer containing TUJ1^+^ cells at day 41. Scale bars, 50 μm. **(C)** Mean value of the thicknesses of the neuronal zone layer (μm). Three measurements were performed for each zone in each structure. (*n* = independent experiments; healthy control: *n* = 10 and 19 cortical structures from at least 10 individual organoids; IC-MeCP2:R255X: *n* = 5 and 14 cortical structures from at least 10 individual organoids; MeCP2:R255X: *n* = 4 and 25 cortical structures from at least 10 individual organoids). **(D)** Representative immunofluorescence images of KI67 proliferative marker and TUJ1^+^ neuronal marker at day 41. Scale bars, 50 μm. **(E)** Quantification of the% of KI67^+^ cells as determined by flow cytometry analysis. **(F)** qRT-PCR analysis of the expression of **(i)** the Notch target and the transcription repressor of proneuronal genes *HES5* (days 41) and **(ii)** the Notch ligand *DLL1* (day 41). mRNA levels relative to *GAPDH* and normalized to the control condition. (*n* = independent experiments; WT: *n* = 6; IC-MeCP2:R255X: *n* = 4; MeCP2:R255X: *n* = 3). **(G)** Representative images of *in situ* hybridization of **(i)** *HES5* and **(ii)** *DLL1* as RNA probes and their co-localization with TUJ1 performed by Immunofluorescence. Scale bars, 50 μm. **(H)** Representative immunofluorescence images of HES5 staining and the co-localization with the progenitor marker SOX2. Scale bars, 10 μm. For all graphics depicted, Student’s *t*-test (two-tailed) statistics, was applied *p < 0.05, **p < 0.01, ***p < 0.001; error bars represent SEM.

Considering the imbalance in the neurogenesis process revealed by the female RTT dorsal forebrain organoids, we next questioned which neurodevelopmental processes could be responsible and/or affected by this phenomenon. Quantitative analysis of the thickness of the early born TUJ1^+^ neuronal layer (schematic view in Figure 2A), localized above the SOX2^+^ VZ layer apical region, revealed a significant increase in RTT female organoids (39.03 ± 3.35 μm) in comparison with healthy-control (27.71 ± 2.87 μm) and IC organoids (24.22 ± 2.10 μm) (Figures 2B,C). Moreover, flow cytometry analysis revealed a significantly lower percentage of KI67^+^ proliferative cells in female RTT dorsal organoids (24 ± 1%) in comparison with the control (37 ± 4%) and the IC organoids (35 ± 8%) (Figure 2E and Supplementary Figure 2C). In agreement with these results, histological sections of dorsal organoids revealed the presence of KI67^+^ cells in control and IC organoids, mainly located near the apical surface of VZ-area, whereas a lower number of these cells were observed in RTT organoids (Figure 2D). We also examined cell death by apoptosis as a potential mechanism for the undetectable IP cell pool in female RTT organoids by quantifying the cells expressing cleaved (active) caspase-3 (CAS3). However, this analysis revealed the presence of rare apoptotic cells, at day 41, for all the organoids (Supplementary Figures 2A,B). Altogether, these results suggest a possible imbalance in the generation of differentiating neurons in RTT organoids, with premature exhaustion of proliferating progenitor pool and increase in the number of newborn neurons.

Notch signaling regulates the generation and maintenance of several cell types, including cells from the SVZ, outer SVZ and cortical plate (Kageyama et al., 2008). To elucidate the molecular mechanisms by which MeCP2:R255X mutation may influence the premature transition toward neuronal differentiation, we examined mRNA levels at day 41 of the Notch ligand Deltalike1 (*DLL1*) and of the Hes family basic helix-loop-helix factor 5 (*HES5*), a direct downstream target of Notch. The levels of *HES5* mRNA were found significantly increased for the female RTT dorsal organoids when compared with the healthy-controls, and increased in comparison to the IC organoids (Figure 2Fi). On the other hand, it was observed a significant decrease in the expression of *DLL1* in MeCP2:R255X organoids (Figure 2Fii). The premature formation of post-mitotic neurons can be related with the dynamics of gene expression of Notch operands that regulates, both cell-autonomously and cell-non-autonomously, the time-transitions of progenitor cells into differentiated neural cells (Kageyama et al., 2008). Thus, for better understanding the levels of gene expression of Notch signaling ligands and targets, we performed *in situ* hybridizations at day 41, co-localized with immunostaining for TUJ1^+^ neurons and SOX2^+^ progenitors. In healthy-controls and IC organoids the expression of the Notch ligand *DLL1* is observed in the TUJ1^+^ neuronal cell layer, and also in scattered progenitors (Figure 2Gii). On the other hand, in female RTT organoids, *DLL1* expression is reduced, being expressed exclusively in TUJ1^+^ post-mitotic neurons (Figure 2Gii). Furthermore, *HES5* expression is found only in PAX6^+^ progenitor cells (Supplementary Figure 2D), and not in the post-mitotic TUJ1^+^ neurons (Figure 2Gi), as it would be expected. Moreover, the expression of the HES5 protein is also higher in the female RTT organoids when compared with controls (Figure 2H). Altogether, these results suggest a possible deregulation of the Notch signaling in MeCP2:R255X organoids, during the stage of deep cortical layer formation.

### MeCP2:R255X Neurons Exhibit Altered Intracellular Calcium Dynamics and Functional Neural Network

**FIGURE 3:**
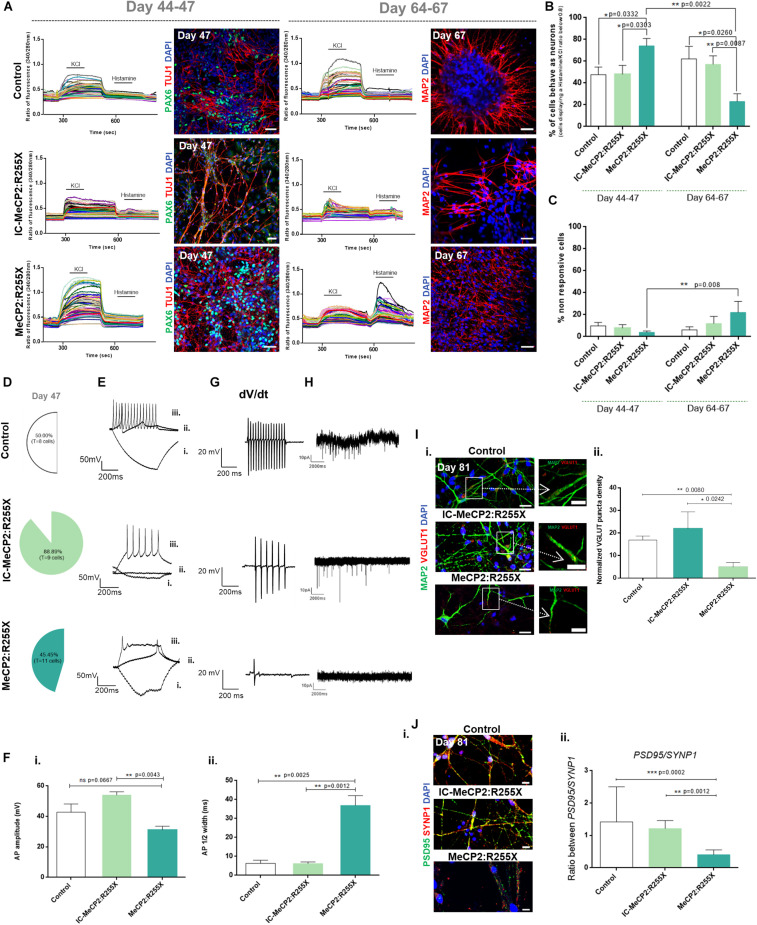
Functional analysis of dorsal forebrain organoids during maturation. **(A–C)** Single cell calcium imaging (SCCI) of dorsal organoids. **(A)** Representative fluorescence ratio profiles (left) of individual cells on days 44–47 and 64–67. Representative immunofluorescence images (right) for the neural progenitor marker PAX6 and for the new-born neuronal marker TUJ1 on days 44–47 and for the mature neuronal marker MAP2 on days 64–67. Immunofluorescence analysis was performed by using the cultures previously analyzed by SCCI. Scale bar 50 μm; **(B)** Percentage of cells displaying a Histamine/KCl ratio below 0.8, which is indicative of the% of cells functionally behaving like neurons. **(C)** Percentage of cells that neither respond to histamine neither to KCL. Total number of cells analyzed (*n* = independent experiments): Control: *n* = 6 with 618 cells analyzed at day 44–47 and 407 cells analyzed at days 64–67; IC-MeCP2:R255X: *n* = 4 with 358 cells analyzed at days 44–47 and 271 cells analyzed at days 64–67; MeCP2:R255X: *n* = 3 with 461 cells analyzed at day 44–47 and 415 analyzed at days 64–67. **(D–G)** Patch-clamp recordings of single cells at day 47. **(D)** Percentage of cells depicting at least one firing action potential (AP). **(E)** Representative traces of firing responses evoked under current-clamp mode by injection of a 500 ms current pulse [–25 **(i)** to +275 pA in 25-pA increments **(ii,iii)** from an initial holding potential (Vh) of –70 mV]. Scale bars correspond to 50 pA and 200 ms. **(F) (i)** AP amplitudes measured from the first AP depicted; **(ii)** Half-peak width measured from the first AP depicted. **(G)** Representative views of the AP velocity firing near threshold (dV/dt). Scale bars correspond to 20 pA and 200 ms. **(H)** Representative recordings of spontaneous postsynaptic currents. Scale bars correspond to 10 pA and 2000 ms. **(I) (i)** Representative immunofluorescence images of 4–5 days-old replated organoid cultures stained for VGLUT1 and MAP2. Scale bar 20 μm. Representative amplification of neurons showing VGLUT1 puncta on MAP2 neurites. Scale bar 10 μm. **(ii)** Normalized density of VGLUT1 pre-synaptic puncta. *n* = independent experiments; Control: *n* = 7; IC-MeCP2:R255X: *n* = 4; MeCP2:R255X: *n* = 6. **(J) (i)** Immunofluorescence images of PSD95 and SYNP1. The overlap between PSD95 and SYNP1 (yellow) indicate sites of synaptogenesis. Scale bar 10 μm. **(ii)** PSD95/SYNP1 ratio measured from immunofluorescence images. For all graphics depicted, Student’s *t*-test (two-tailed) statistics was applied: *p < 0.05, **p < 0.01, ***p < 0.001; error bars represent SEM.

The functionality of mature neurons derived from dorsal organoids, generated from healthy-controls, IC and RTT female hiPSC lines, was characterized using single cell calcium imaging (SCCI) and whole-cell patch-clamp analysis (Silva et al., 2020). SCCI analysis was used for functional discrimination of the organoid populations in terms of expression/sensitivity of voltage sensitive calcium channels (VSCC) and for testing their responsiveness to depolarization. Functional recordings of SCCI were performed at days 44–47 and at days 64–67, during which time cultures were maintained in serum-free BrainPhys^TM^ medium (Satir et al., 2020) (Supplementary Figure 3A). Dorsal organoids were preloaded with the calcium indicator Fura-2AM on the day of the experiment. Cells were then exposed to 50 mM of KCL, which in excitable cells, like neurons, leads to the opening of VSCCs and a massive influx of calcium, followed by stimulation with Histamine, which leads to an increase in intracellular calcium concentration in stem/progenitor cells due to the activation of H1 receptor (Molina-Hernández and Velasco, 2008). Figure 3A shows representative peaks of SCCI for replated organoids obtained during KCL and Histamine stimulation. Histamine/KCl ratios for mature neurons are typically below 0.8 (Agasse et al., 2008; Bernardino et al., 2013). Between days 44–47, female RTT organoids contained 74 ± 7% of cells behaving like neurons, while the mean percentage of neurons present in healthy-controls and IC organoids was 48 ± 7% and 48 ± 8%, respectively. At later time points, between days 64 and 67, an expected maturation was observed in healthy-controls and IC organoids, with 62 ± 11% and 57 ± 8% of cells presenting a neuron-like response, respectively (Figure 3B). However, MeCP2:R255X organoids displayed a significant decrease in the percentage of cells with neuron-like response in comparison with days 44–47 of differentiation, and also in comparison with control organoids. Immunostaining analysis revealed that, on days 44–47, female RTT organoids exhibited denser TUJ1^+^ neuronal networks compared with IC and healthy controls and on days 64–67, MeCP2:R255X neurons showed punctate MAP2 staining (Figure 3A). Nevertheless, from days 44–47 to 64–67, this culture showed a significant increase of cells not responsive to KCL neither to Histamine (Figure 3C). Thus, altogether, these results indicate that prematurely differentiated MeCP2:R255X neurons might not be able to complete their functional maturation.

Functional maturation of dorsal organoids was also assessed by whole-cell patch-clamp, at days 44–47. For the healthy-control organoids, a total of 8 cells were analyzed, with 50% of the cells being able to respond to a continuous current injection, demonstrating a typical repetitive neuronal firing action potential (AP) (Figures 3D,E). For the IC organoids, from a total of 9 cells analyzed, 88% presented a typical firing AP. However, for the MeCP2:R255X organoids, from a total of 11 cells, 45% exhibited an atypical firing AP, with a decreased firing frequency (Supplementary Figure 3Bi) and with most of the neurons presenting an abortive-like AP (Figures 3D,E). Overall, the majority of control neurons exhibited more mature AP firing properties, presenting a faster and consistent AP velocity near threshold (dV/dt), while MeCP2:R255X neurons were more immature, presenting a reduced AP velocity (Figure 3G).

Additionally, from the analysis of spiking cells, a significant reduction in AP amplitude was observed for MeCP2:R255X organoids when compared with IC, and also when compared with the healthy-control neurons (Figure 3Fi). As shown in Figure 3Fii, the half-peak width of female RTT neurons was significantly larger when compared with that of the control neurons. In spiking cells, the resting membrane potential values were similar for all the neuronal cells analyzed (Supplementary Figure 3Bii). At later time points of maturation, by day 81, healthy-control neurons presented an increase in spiking activity, whereas for the MeCP2:R255X neurons, none of the 8 cells analyzed presented firing AP (see Supplementary Figures 3C,D). Moreover, spontaneous currents, which provide an indication of the presence of functional neurotransmitter receptors, were also recorded. The representative traces indicate the absence of synaptic transmission for MeCP2:R255X organoids and the establishment of some neuronal functional connections for both the control and the IC-derived neurons (Figure 3H).

Neurons from dorsal organoids were also characterized concerning the expressing of characteristic markers of mature neurons. A higher mRNA expression of the vesicular glutamate transporter marker VGLUT1 was observed at day 81 in MeCP2:R255X organoids when compared with all control neurons (Supplementary Figure 3E), with VGLUT1 puncta being observed in MAP2+ dendrites (Figure 3Ii). However, a reduction in the density of VGLUT1 puncta was observed in MeCP2:R255X organoids when compared with control and IC organoids (Figure 3Iii).

Furthermore, cells were stained for the pre-synaptic protein synapsin-1, SYNP1, and the post-synaptic-protein PSD95 and the PSD95/SYNP1 ratio was found to be significantly lower for RTT organoids, which could indicate a decreased synaptic density (Figure 3Jii). Additionally, the co-localization of these two indicators of mature excitatory synapses was observed in healthy-control and IC organoids but not in female RTT neurons (Figure 3Ji). Interestingly, we observed the presence of GFAP^+^ astrocytes in close association with neurons in all the dorsal organoids at day 81 (Supplementary Figure 3F).

Ca2^+^ diffusion and synaptic transmission have been correlated with spine morphology, which changes during developmental maturation (Risher et al., 2014). As MeCP2 has been proved to be essential for dendritic spine formation, the spine head width and neck length of neurons in dorsal organoids were measured at days 47 and 67. The proportion of mushroom and stubby spines, which increases with spine complexity and maturation, was found to decrease along the differentiation for the female RTT organoids, which instead presented an increase in the number of filopodia and long thin spine types. Conversely, an increased number of more mature branched spines were observed in all control neurons (see Supplementary Figures 3G,H).

Taken together, these results indicate that healthy-control and IC dorsal organoids supported the generation of functional neurons, by establishing neuronal connectivity, while MeCP2:R255X organoids present defects in neuronal maturation.

### Impaired Interneuron Migration in RTT Forebrain Organoids

**FIGURE 4:**
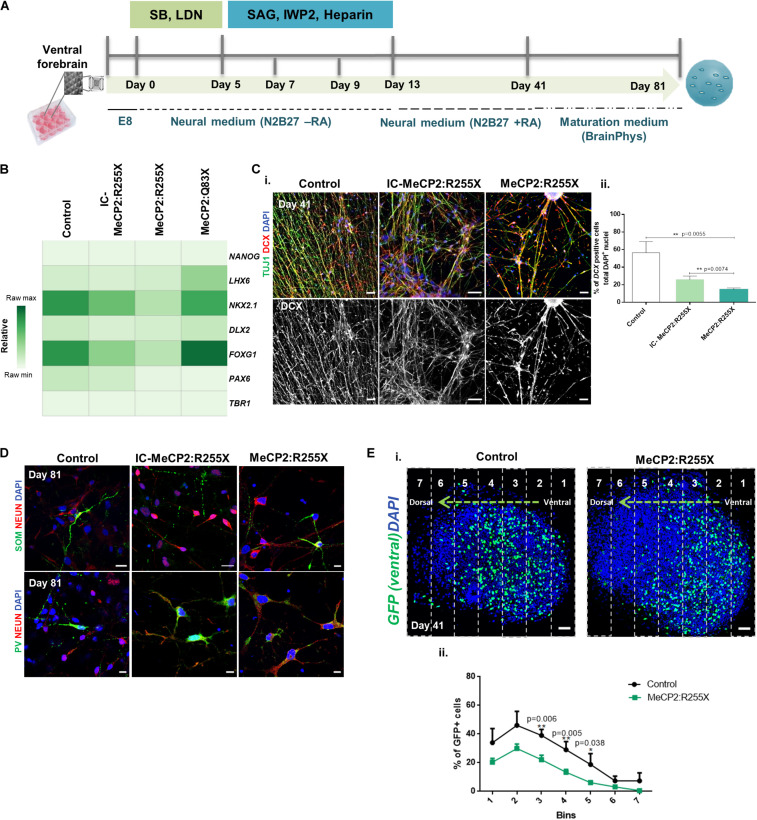
Ventral and fused forebrain organoids derived from the female patient-specific RTT hiPSC line reveal impairments in cell migration. **(A)** Schematic overview of the protocol for ventral patterning of hiPSCs until day 81 of culture. **(B)** Transcription profile of ventral forebrain organoids at day 41 of differentiation. Quantitative RT-PCR analysis for the pluripotency marker *NANOG*, the forebrain ventral markers *LHX6, NKX2.1* and *DLX2*, the generic forebrain marker *FOXG1* and the dorsal markers *PAX6* and *TBR1*. All values were normalized to *GAPDH*. Raw minimum (min) and raw maximum (max) values were taken as a reference for the heatmap representation. *n* = independent experiments; Control: *n* = 11; IC-MeCP2:R255X: *n* = 4; MeCP2:R255X: *n* = 4; MeCP2:Q83X: *n* = 5. **(C) (i)** Immunofluorescence images of replated ventral organoids stained for DCX and TUJ1 (images used for DCX count). Scale bar 50 μm **(ii)** Percentage of DCX^+^ immature migrating interneurons relative to total DAPI^+^ nuclei. *n* = independent experiments; Control: *n* = 3 (5 images); IC-MeCP2:R255X: *n* = 3 (9 images); MeCP2:R255X: *n* = 3 (11 images). **(D)** Immunofluorescence staining of replated ventral organoids for markers of MGE-derived GABAergic neurons, PV and SOM, with the mature neuronal marker NEUN. Scale bar 10 μm. **(E) (i)** Fused organoids (GFP^–^ dorsal organoids fused with GFP^+^ ventral organoids) stained with DAPI. Images were divided into 7 segments, the % of GFP^+^ cells was analyzed in each segment and normalized to the DAPI^+^ nuclei. Scale bar 50 μm **(ii)** Migration distance for GFP^+^ cells in MeCP2:R255X derived-fused organoids. Control (9 organoids); MeCP2:R255X (9 organoids). For all graphics depicted, Student’s *t*-test (two-tailed) statistics, was applied *p < 0.05, **p < 0.01; error bars represent SEM.

Ventral cortical organoids were generated from all hiPSC lines and cultured until day 81 (Figure 4A). Neural induction was performed by dual-SMAD inhibition followed by activation of the sonic hedgehog (SHH)-signaling pathway, as previously described (Bagley et al., 2017). The ventral identity of organoids was assessed by qRT-PCR analysis at day 41 (Figure 4B). The mRNA expression of typical dorsal markers, *TBR1* and *PAX6*, and of the pluripotency marker *NANOG* was almost undetectable. The ventral marker *DLX2* was expressed at low levels, accompanied with high expression of the forebrain marker *FOXG1* (Figure 4B). qRT-PCR was also used to characterize the expression of the different interneuron progenitor’s subtypes that are produced in specific subregions of the ganglionic eminence (GE) zone of the ventral forebrain (Gelman et al., 2012). *NKX2.1*, which is expressed in the MGE (Sandberg et al., 2016) and from which several ventral interneuron subtypes are generated, was found highly expressed in our ventral organoids. The mRNA levels of *LHX6*, expressed in a sub-region of MGE that also originates ventral interneuron subtypes, was also found increased in ventral organoids, with no significant differences being found between controls and RTT organoids (Figure 4B). Importantly, at day 41, RTT cells showed a significant decrease in mRNA levels for the *NKX2.1* gene when compared with the healthy control cells, and a tendency for a decreased expression of this marker in comparison with the IC organoids (Supplementary Figure 4A). Again, no significant differences were observed between male ventral RTT organoids and the respective healthy controls, so we decided to proceed with a detailed analysis for the RTT female cell line.

MGE-derived post-mitotic interneurons migrate toward the dorsal cortex region, integrating into an appropriate neuronal circuit (Zimmer-bensch, 2018). To investigate potential alterations to this process in female RTT ventral organoids, the expression of DCX, a marker for immature migrating interneurons, was quantified at day 41. Interestingly, the expression levels for DCX were significantly decreased in the ventral female RTT organoids, when compared with all control organoids (Figures 4Ci,ii). It is also known that the large majority of neurons produced in the MGE region express parvalbumin (PV) and somatostatin (SOM) (Gelman et al., 2012). The presence of SOM^+^ and PV^+^ interneurons at day 81 was indeed detected in all our ventral organoids (Figure 4D). The possible alteration in the generation of *NKX2.1^+^* interneuron progenitors and the observed DCX decrease could consequently induce changes in the interneuron migratory dynamics. To evaluate this hypothesis, we employed a more complex system, obtained by assembling organoids of dorsal and ventral forebrain identities (Bagley et al., 2017; Xiang et al., 2017) (Supplementary Figure 4B). Upon characterization of the fused organoids at day 41, we found the same molecular alterations previously observed in independently generated dorsal and ventral organoids regarding the expression of *TBR1* and *NKX2.1* (see Supplementary Figures 4Ci,ii). To follow the process of interneuron migration, the MECP2:R255X and two control cell lines were induced to express GFP by lentiviral transfection. To test whether interneurons from the ventral region could migrate, the ventral patterning protocol using the GFP^+^ reporter line and the dorsal patterning protocol using the GFP^–^ cell line were employed. The assembly of both organoid types was then performed at day 13, with merged organoids being observed already by day 14 (Supplementary Figure 4D). Finally, we analyzed migrating GFP^+^ cells at day 41 in organoid cryosections. Interestingly, it could be observed that the GFP^+^ ventral cells in MECP2:R255X fused organoids migrated shorter distances than GFP^+^ cells from healthy-control hiPSCs (Figures 4Ei,ii). These fused organoids derived from the controls and MECP2:R255X lines were later characterized, at day 81, by immunostaining. It was found that they contained cells expressing MAP2 and the astrocyte marker GFAP (Supplementary Figure 4Ei), as well as the mature glutamatergic pyramidal neuronal marker VGLUT1 and the GABAergic interneuron marker VGAT (Supplementary Figure 4Eii.).

## Discussion

Herein, hiPSCs derived from RTT patients were used to model the development of the human forebrain by using region-specific and assembled forebrain organoids. Several aspects were studied, including the formation of the neuroepithelial layers, their intrinsic cellular diversity and the neuronal network functionality. In contrast with the majority of MeCP2 gene-KO mice and hiPSCs-based RTT models, we developed 3D models presenting phenotypical characteristics of RTT caused by the female MeCP2:R255X mutation and the male MeCP2:Q83X mutation, present in the human population.

First, we developed organoids exhibiting the main molecular developing features of the dorsal forebrain domain. MeCP2:R255X dorsal organoids displayed an increase in gene and protein expression for the TBR1 marker, present in glutamatergic post-mitotic neurons in the cortical subplate (Englund et al., 2005). Our data also indicate a lack of TBR2^+^ IPs in these organoids that might be caused by the increased levels of TBR1. In fact, TBR2 downregulation occurs upon upregulation of TBR1, and upon formation of early born neurons that migrate through the inner zone into the cortical plate (Englund et al., 2005). Importantly, mouse models showing *TBR2* downregulation have been associated to microcephaly (Arnold et al., 2008), with the decreased brain volume accompanied by increased neuronal density, a feature also reported in RTT patients (Schanen et al., 2004) and RTT mouse models (Chen et al., 2001). Consistently, our results show a clear correlation between the formation of the early post-mitotic cortical deeper layers and an increase in neuronal layer thickness, and a decrease of KI67^+^ proliferative cells. Additionally, a decreased expression of PAX6 mRNA and a lower number of HOPX^+^ oRG cells observed could be a possible evidence of the imbalance between neuronal differentiation and maintenance of the progenitor pool.

Our RTT male dorsal organoids did not reveal the same initial phenotypic alterations observed in the female ones, further demonstrating the potential capacity of our models to highlight mutation-dependent alterations in the differentiation/proliferation equilibrium.

In line with our findings, Li et al. (2014) found that mutant-*MeCP2* serine 421 phosphorylation in progenitor cells, isolated from adult mouse hippocampus, displayed an imbalance between proliferation and neuronal differentiation, and that this imbalance was mediated by alterations of the Notch signaling pathway (Li et al., 2014). Moreover, *Edri et al.* verified that neurons populating the cortical deep layers are generated from RG progenitors in a Notch-dependent manner. It was also shown that early progenitor stages require Notch activation to generate early appearing neurons (Edri et al., 2015). Our data shows that the premature appearance of deep cortical neurons observed in MeCP2:R255X dorsal organoids is associated to *HES5* overexpression, which points to the hypothesis that the observed premature differentiation may be a consequence of alterations of the Notch regulatory mechanism. In fact, Notch ligands, effectors and pro-neuronal genes are expressed in an oscillatory, dynamic and site-specific manner in the developing mammalian nervous system (Kobayashi and Kageyama, 2010; Kageyama, 2011). In line with our observations, Bansod et al. (2017) demonstrated that *Hes5*-overexpression in mice induced the early shift from deep to superficial layer neurogenesis and regulated the timing of neurogenesis. As an alternative hypothesis, a regulatory mechanism known as lateral inhibition, which is responsible for the proper maintenance of the pool of neural progenitors during neurogenesis, may be altered. Under normal circumstances, the newborn neurons express the Notch ligand *DLL1*, activating the Notch signaling in the neighboring cells, thus inhibiting their neuronal differentiation (Kageyama et al., 2008). However, when this pathway is deregulated, neural progenitors prematurely differentiate into early born neurons. As the mRNA levels of the Notch ligand *DLL1* were decreased in our RTT female organoids, the premature formation of post-mitotic neurons in these organoids could indicate a deregulation of Notch signaling pathway, more precisely the mechanistic process of lateral inhibition. In fact, the previously mentioned report from Li et al. (2014) also demonstrated that phosphor-mutant MeCP2 protein has altered promoter occupancy at the promoters of *DLL1*, decreasing the levels of transcription of this Notch ligand (Li et al., 2014). Future studies should be performed to confirm our hypothesis of alterations of the Notch signaling lateral inhibition pathway driven by the MeCP2:R255X mutation.

We also studied the impact of mutated *MeCP2* in regulating neural function and activity in dorsal forebrain organoids namely by performing SSCI and patch-clamp analysis. Organoid slices have been commonly used by others (Xiang et al., 2017) for this type of studies namely because this method allows the preservation of the original cell to cell interactions and the maintenance of the 3D organization in the selected part of the slice. However, in the case of our approach, the 3D organoids were dissociated and replated 4–5 days before the analysis because we believe that analyzing networks without dense cell overlaps is a more accurate way to perform the recording and the analysis of both calcium imaging and patch-clamp experiments. Since the organoid forming cells were capable of re-establishing the self-organized neuronal networks upon replating, we believe that this approach allows this functional analysis to capture the interactions between the cells that were previously composing the entire 3D organoid, both neurons and progenitor cells. In addition to SSCI and patch-clamp analysis, also immune quantifications of pre- and pos-synaptic markers, as well as dendritic spines characterization, were performed by replating of the 3D organoids, thus keeping all the manipulation procedures for functional-related analysis and the respective results consistent.

Single cell calcium imaging performed at days 44–47 of differentiation of RTT female dorsal organoids corroborate the previously raised hypothesis of premature development of newborn neurons. Later in differentiation, SCCI analysis showed an increased percentage of cells that do not respond to KCl or histamine stimulation, suggesting a decrease in the percentage of cells presenting a neuronal-like profile along the maturation process. This is consistent with previous studies showing *MeCP2*-mutant neurons with altered calcium signaling (Chen et al., 2003; Marchetto et al., 2010), associated to an impairment in the expression/sensitivity of VSCC and consequently to a defective neuronal responsiveness to depolarization (Gandaglia et al., 2019).

The capacity of human cortical neurons to generate repetitive AP upon a current depolarization was not detected before 20 gestational weeks (GW), whereas single AP firing was present as early as 16 GW (Moore et al., 2009). We observed that neurons in our control dorsal organoids presented features of maturation, exhibiting electrophysiological activity. Conversely, the prematurely formed neurons in MeCP2:R255X dorsal organoids, at day 47, exhibit slow activity, with decreased firing properties. This was demonstrated by the appearance of one single AP firing in almost all of the analyzed cells, by the broad AP half width of the first AP, by the decreased AP amplitude and by the reduced AP velocity (dV/dt) in a train of AP firing near threshold. APs are generated by special types of voltage-gated ion channels embedded in the plasma membrane, such as voltage-gated potassium and sodium channels (Johnson et al., 2007; Weick, 2016; Silva et al., 2020). Thus, our results observed in APs generated by MeCP2:R255X neurons may potentially indicate the presence of fewer release sites or less release probability. Our results are aligned with other studies previously published in the literature. In fact, electrophysiological recordings of RTT hiPSCs-derived neurons had already exhibited immature spike characteristics (Marchetto et al., 2010). Moreover, a different study analyzing RTT neurons showed dysfunction in AP generation and voltage-gated Na^+^ currents (Djuric et al., 2016). Nevertheless, for the exact determination of potential alterations of neuronal membrane ion channels, in the future both sodium and potassium current injections measurements must be performed. As another demonstration of an altered electrophysiological profile, synaptic transmission, which requires the existence of functional neurotransmitter receptors, was also affected in our RTT female organoids, as determined by analysis of spontaneous synaptic currents, suggesting the existence of impairments in neuronal maturation and synaptic connection.

Later in maturation, at day 81, no AP was detected in dorsal MeCP2:R255X neurons. In agreement with this result, our analyses also demonstrated a decrease in VGLUT1 puncta, a specific protein of glutamatergic neurons responsible for loading glutamate into synaptic vesicles. In contradiction with these results, an increase in mRNA levels of *VGLUT1* was detected, which could be potentially related with a homeostatic compensation for the differential number of pre- and post-synaptic structures observed. In fact, previous studies using postmortem samples of RTT female patients demonstrated differential temporal distributions in glutamate levels in the forebrain (Lane et al., 1999). In agreement with our VGLUT1 puncta analysis, other studies using MECP2-mutated neurons derived from hiPSCs (Marchetto et al., 2010) or neurons lacking MECP2 derived from mice (Chao et al., 2007), also revealed a decrease in VGLUT1 puncta and consequently a decrease in functional synaptic densities.

As an additional functional characterization, we analyzed the dendritic spine morphology, as they are known to present a reduced density in RTT patients (Armstrong, 1995; Zhou et al., 2006). Accordingly, we observed a reduction of the number of mushroom and stubby-shaped spines in our RTT neurons, already reported for other RTT models (Chapleau et al., 2009).

Overall, our RTT female organoids revealed functional immaturity/impairments, excitatory imbalance and regression in spine morphology. This can be potentially explained by the premature formation of early post-mitotic neurons that possibly lack the spatial-temporal cues needed for a proper maturation. Moreover, together with other studies reported in the literature (Blue et al., 1999; Marchetto et al., 2010), our results suggest an apparent role of MeCP2 in regulating glutamatergic synapse formation, synaptic function and neuronal maturation features.

Upon ventral patterning of RTT hiPSCs, we observed a significant decrease in the expression of the MGE domain characteristic marker, *NKX2.1*, in RTT female ventral organoids. MGE domain is composed of progenitor cells that originate GABAergic interneuron subtypes. Thus, this result is consistent with previous studies using *MeCP2*-mutant mice in which GABA-releasing neurons showed reduced inhibitory neurotransmitter capacity, being responsible for RTT symptoms such as repetitive behaviors and impaired motor coordination (Chao et al., 2010). During neurodevelopment, interneurons formed in MGE begin a migratory process to populate the dorsal pallium, by performing a tangential migration followed by integration into neural circuits, undergoing activity-dependent maturation (Gelman et al., 2012). A study from Sandberg et al. (2016) demonstrated that the combinatorial binding of *NKX2.1* and *LHX6* promotes the transcriptional activation of genes expressed in cortical migrating interneurons (Sandberg et al., 2016), influencing the process of interneuron migration. Moreover, *Nkx2.1* mutant-mice demonstrated deficits in the MGE migration (Anderson et al., 2001). Consistently, we also found a decrease of DCX expression for ventral MECP2:R255X organoids. In fact, previous studies in *DCX*-KO-mouse models demonstrated that this microtubule-associated protein is crucial for radial migration of interneurons (Liu et al., 2007), affecting their number and distribution (Kappeler et al., 2006). In a more recent study using 3D organoids and monolayer RTT-derived *MeCP2*-knockdown hiPSCs, it was also observed a decrease in DCX expression, and consequently defects in neurogenesis and neuronal differentiation (Mellios et al., 2017).

Taking together these alterations of the molecular and functional profile of interneurons, we decided to develop a more appropriate model to study their migration process by assembling ventral and dorsal organoids (Bagley et al., 2017; Xiang et al., 2017; Sloan et al., 2018). By using this model we observed that neurons traveled a shorter distance during their migration process when using female RTT fused organoids and we also observed a decreased percentage of GFP^+^ ventral cells integrating into the dorsal part of the assembloid at early stages of development. So far, no other studies were yet performed regarding interneuron migration in the context of RTT. However, it is known that migratory interneurons integrate into neural dorsal circuits, contributing to the E/I balance (Gelman et al., 2012), whose disturbance has been previously reported for RTT (Chao et al., 2010). It is known that neuron-specific K^+^ – Cl^–^ co-transporter 2 (KCC2) is a critical downstream gene target of *MeCP2*, being responsible for neuronal network formation, maturation delay and perturbations of GABAergic neurotransmission (Tang et al., 2016; Ruffolo et al., 2020). It was also proved that signaling molecules such as GABA neurotransmitter, influenced neuronal progenitor proliferation and embryonic neuron migration (Wang et al., 2003). In the future, our fused organoid model may be treated with the active peptide fragment of Insulin-like Growth Factor 1 (IGF-1) (Tropea et al., 2009), in order to study the possible rescue of neuronal migration and network integration delays, and consequently the rescue of functional GABA deficits. Additionally, efforts should be made to further identify which specific subtypes of interneurons are affected. The disequilibrium between excitation and inhibition (E/I) in RTT could be a consequence of both glutamatergic neurotransmission deregulation and GABAergic neurotransmission defects. Our fused forebrain organoids, resembling neural organization, differentiation and network formation of both dorsal and ventral regions, and containing both GABAergic and glutamatergic neuronal sub-types, could be intensively used for the study of the nature and mode of action of the local signals responsible for the (E/I) disequilibrium of different RTT patients.

## Conclusion

Our model highlights the impact of MeCP2 mutations in different stages and brain regions during forebrain development. We have observed previously described aspects of the disease, particularly for later stages, which have been reported for both hiPSC-derived cells and animal models, suggesting the relevance of our platform for this type of studies. However, the model also demonstrates that different mutations are associated to distinct phenotype characteristics, as revealed by the results obtained with two different RTT cell lines. Importantly, our model revealed early developmental alterations in one female RTT-derived cell line, which have not been revealed before using other models, probably because our platform provides an improved recapitulation of the initial stages of cortical layer formation. Overall, we were able to show that our RTT forebrain organoid models can greatly help toward a better understanding of the pathophysiology of RTT in a patient-specific context, and for further testing of potential candidate drugs or other therapeutic strategies in RTT patient-specific organoids. An added value of our investigation relies on the fact that all the significant differences observed are based on comparisons performed between two hiPSC lines with the same genetic background (isogenic pair). In the future, our conclusions must be confirmed by extending our work to studies with other cell lines of the same patient and additional cell lines from other patients carrying the same mutation. Nevertheless, we truly believe that our results are reliable and that our work at this stage is already a valuable and innovative contribution to the field.

## Data Availability Statement

The raw data supporting the conclusions of this article will be made available by the authors, without undue reservation.

## Author Contributions

All the authors contributed to design research. AG performed the hiPSCs expansion and differentiation cultures and cell culture characterization. TF and TS assisted with cell culture maintenance, growth and characterization. TF assisted with flow cytometry experiments. EB assisted with *in situ* hybridization and sequencing. SV, SX, and AS contributed for the design of functional experiments. AG and SV performed the single cell calcium imaging and patch-clamp experiments. SD derived the female RTT hiPSCs lines. AG and MD wrote the manuscript. All authors contributed to the article and approved the submitted version.

## Conflict of Interest

The authors declare that the research was conducted in the absence of any commercial or financial relationships that could be construed as a potential conflict of interest. The handling editor declared a shared affiliation with several of the authors, AG, TF, SV, TS, EB, SX, SD, AS, JC, and MD, at the time of review.

## Footnotes

1 http://n2t.net/addgene:12259
