# Supplementary material for: Modeling Rett Syndrome With Human Patient-Specific Forebrain Organoids
Source: Front Cell Dev Biol. 2020 Dec 10;8:610427. doi: 10.3389/fcell.2020.610427 (PMC7758289; doi:10.3389/fcell.2020.610427)
Supplement: Supplementary file 1 [file Data_Sheet_1.PDF]

Supplementary Material

Supplementary Materials and Methods

Aggregate size analysis

During the hiPSC neural induction process, several images were taken at days 0, 2 and 5 for monitoring the size of cell aggregates. Images were acquired using a Leica DMI 3000B microscope with a Nikon DXM 1200F digital camera. The aggregate diameter was calculated using Fiji software (for ImageJ).

Supplementary list of antibodies used

Primary antibodies: PAX6 (rabbit, 1:400, Covance), NESTIN (mouse, 1:200, R&D), TBR2 (rabbit, 1:200, Abcam), SOX2 (rabbit, 1:200, Sigma), SOX2 (mouse, 1:200, Abcam), TBR1 (rabbit, 1:400, Millipore), CTIP2 (rat, 1:200, Abcam), OTX1/2 (rabbit, 1:400, Millipore), NCAD (mouse, 1:1000, BD Transduction), KI67 (mouse, 1:100, Abcam), TUJ1 (mouse, 1:1000, Biolegend), HES5 (mouse, 1:100, Santa Cruz), CAS3 (rabbit, 1:400, Cell Signaling), HUCD (rabbit, 1:200, Thermofisher Scientific), MAP2 (mouse, 1:500, Sigma), VGLUT (rabbit, 1:100, Abcam), PSD95 (mouse, 1:200, Sigma), SYNP1 (rabbit, 1:200, Abcam), GFAP (rabbit, 1:200, Abcam), DCX (rabbit, 1:200, Abcam), PV (mouse, 1:200, Sigma), SOM (mouse, 1:200, Santa Cruz), NEUN (rabbit, 1:200, Cell Signaling), HOPX(mouse, 1:200, Santa Cruz) and VGAT (mouse, 1:500, Santa Cruz).

Secondary antibodies: goat anti-mouse IgG or IgM and goat anti-rabbit IgG, Alexa Fluor®–488 or –546, goat anti-rat IgG and Alexa Fluor®–488 (1:500, Thermofisher Scientific).

Quantification and classification of immunofluorescence images

The immunofluorescence images acquired using the Zeiss LSM 710 Confocal microscope were quantified for the number of TBR1, TBR2 and CAS3 positive cells using the Fiji® software (for ImageJ). The original images were converted into 8-bits, followed by adjustment of a threshold, watershed treatment, and definition of parameters for particle analysis. The total of cell nuclei were counted using DAPI images. The positive stained nuclei were counted and the ratio between positive cells and the total number of cells was calculated. The same previously described approach was used for VGLUT1 puncta density measurements observed in MAP2 positive dendrites. For the PSD95 and SYNP1 staining, the previous approach was used, but instead of performing normalization with the total number of cells, the ratio between PSD95/SYNP1 was determined.

The measurements of the thicknesses of the neuronal layers were performed using ImageJ software. The outer layer was defined by TUJ1 immunoreactivity, which is the area outside the VZ ($SOX2^+$) to the nearest pial surface. Three measurements were performed for each zone, and the respective mean was determined, as indicated in Figure 2A.

For the dendritic spine classification, after phalloidin-staining, cell images were acquired using Zeiss LSM 710 Confocal Laser Point-Scanning Microscopes, with 63x Plan-Apochromat oil objective. Representative images were used to manually measure dendritic spine head, width and neck length using Fiji® software (for ImageJ). Dendritic spines were classified as follows: Filopodia (length > 2µm), Long thin (length < 2µm), Thin (length < 1µm), Stubby (length/width ratio < 1, Mushroom (width > 0.6µm), and Branched (2 or more heads)[29].

For the DCX^+ dendritic quantification, a plugin of Fiji® software (for ImageJ), NeuronJ, was used to facilitate the tracing and analysis of elongated image structures, such as neuronal processes. The traces were identified semi-automatically, quantified in ImageJ and normalized with the total number of cell nuclei, using DAPI images.

The quantification of GFP^+ cells for studying of cell migration was performed by dividing each cryo sectioned image into seven sections with the same dimensions. Section 1 was always defined at the start of the GFP^+ region of the fused organoid. The total number of GFP^+ cells was normalized with the total number of DAPI cells identified in each section. The distribution of GFP^+ cells that moved along with the fused organoids was determined and graphically represented from several images.

Supplementary list of primers used

Taqman® Gene Expression Assays (20X, Applied Biosystems): *NANOG* (HS02387400-g1), *PAX6* (HS00240871-m1), *TBR1 (*Hs00232429_m1), *DLX2 (*HS00269993_m1), *NKX2.1 (*HS00968940_m1), *LHX6 (*HS00232660_m1), *FOXG1 (*HS01850784_s1) and *GAPDH* (HS02758991-g1).
SYBR Green: *DLL1* (fw: TGGCGCAGGCATCGA; rv:GGCGGCTGATGAGTCTTTCT), *HES5* (fw:TCCTGGAGATGGCTGTCAGCTA;rv:CGTGGAGCGTCAGGAACTGCA), *VGLUT1* (fw:TACACGGCTCCTTTTTCTGG; rv:CTGAGGGGATCAGCATGTTT) and *GAPDH* (fw:GAGTCAACGGATTTGGTCGT; rv:TTGATTTTGGAGGGATCTCG)

Supplementary Figures

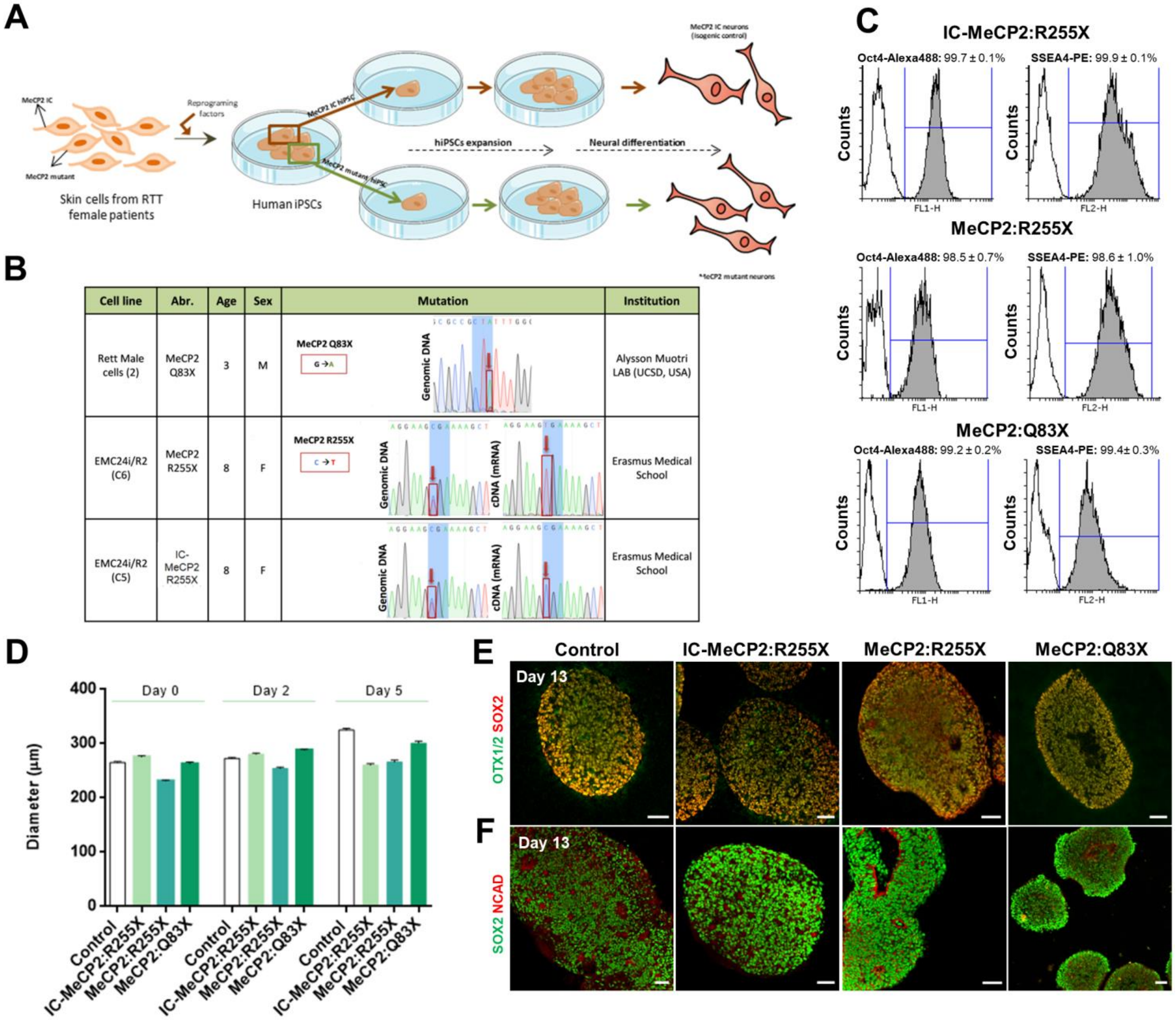

Cell line	Abr.	Age	Sex	Mutation	Institution
Rett Male cells (2)	MeCP2 Q83X	3	M	MeCP2 Q83X G→A	Alysson Muotri LAB (UCSD, USA)
EMC24i/R2 (C6)	MeCP2 R255X	8	F	MeCP2 R255X C→T	Erasmus Medical School
EMC24i/R2 (C5)	IC-MeCP2 R255X	8	F		Erasmus Medical School

Supplementary Figure 1.1. Characterization of RTT patient-derived hiPSCs, hiPSC-derived size-controlled aggregates until day 5 of neural induction and dorsal forebrain patterned organoids at day 13 of culture. A) Schematic overview of the process of derivation of the isogenic cell line from reprogrammed hiPSCs derived from the RTT female patient. **B)** Patient characteristics and sequencing profile of the RTT female MeCP2:R255X, IC-MeCP2:R255X and male MeCP2:Q83X hiPSC lines, exhibiting the mutations in genomic DNA and in the active X chromosome by cDNA sequencing. Genetic screening of the EMC24i/R2 patient revealed the heterozygous single-nucleotide mutation C→T. The RTT male hiPSC line exhibited the single nucleotide mutation G→A, responsible for the premature stop codon. For the IC and MeCP2:R255X hiPSCs line, sequencing was performed from cDNA of differentiated cells at day 41, from each independent differentiation experiment. **C)** Representative histograms of the flow cytometry analysis confirming the pluripotency of RTT female MeCP2:R255X, IC and male MeCP2:Q83X hiPSC lines by quantification of the percentage of cells expressing OCT4 and SSEA4. n=3 independent experiments were performed for each cell line. **D)** Estimation of aggregate size (days 0, 2 and 5)

during hiPSC induction towards neuroectodermal fate as size-controlled aggregates. The induction was started with size-controlled aggregates with diameter between 250-300µm. No statistical analysis was applied. **E)** Immunofluorescence analysis of aggregate slices at day 13 after initiation of neural induction, for OTX1/2 and SOX2 neural progenitors. **F)** Representative images of neural rosettes on aggregate slices stained for NCAD and SOX2 also at day 13. Scale bars, 50µm

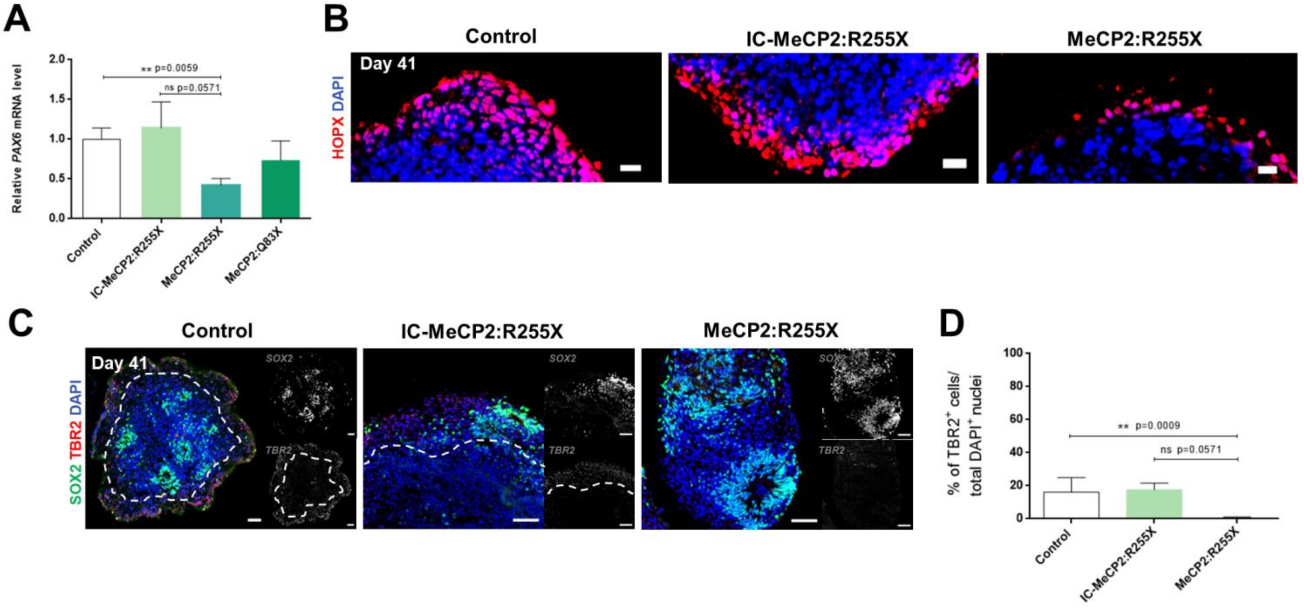

Supplementary Figure 1.2. Altered *TBR2* and *PAX6* expression in female RTT dorsal forebrain organoids. A) Relative mRNA levels of *PAX6* on dorsal organoids at day 41 of differentiation already represented on the heatmap of **Figure 1C**. mRNA levels relative to *GAPDH* and normalized to control condition. n=independent experiments; Control n=10; WT-MeCP2:R255X n=5; MeCP2:R255X n=4; MeCP2:Q83X n=4. For all graphics depicted, student´s t-test (two-tailed) statistics, was applied * p<0.05, ** p<0.01, *** p<0.001; error bars represent SEM. **B)** Immunofluorescence representative images of dorsal organoid sections stained against HOPX (specific molecular marker for the oRG cells from the prominent oSVZ layer). Scale bars, 20µm **C)** Representative images of dorsal organoid sections stained against SOX2 (neural progenitor marker) and TBR2 (IP cell marker). Scale bars, 50µm. **D)** Immunocytochemistry quantification of the percentage (%) of cells expressing TBR2 normalized to the total number of cells stained with DAPI. TBR2: Control n=3 (9 organoids); IC-MeCP2:R255X n=3 (5 organoids); MeCP2:R255X n=3 (6 organoids). n=independent experiments. For all graphics depicted, student´s t-test (two-tailed) statistics, was applied *p<0.05, ** p<0.01, *** p<0.001; error bars represent SEM.

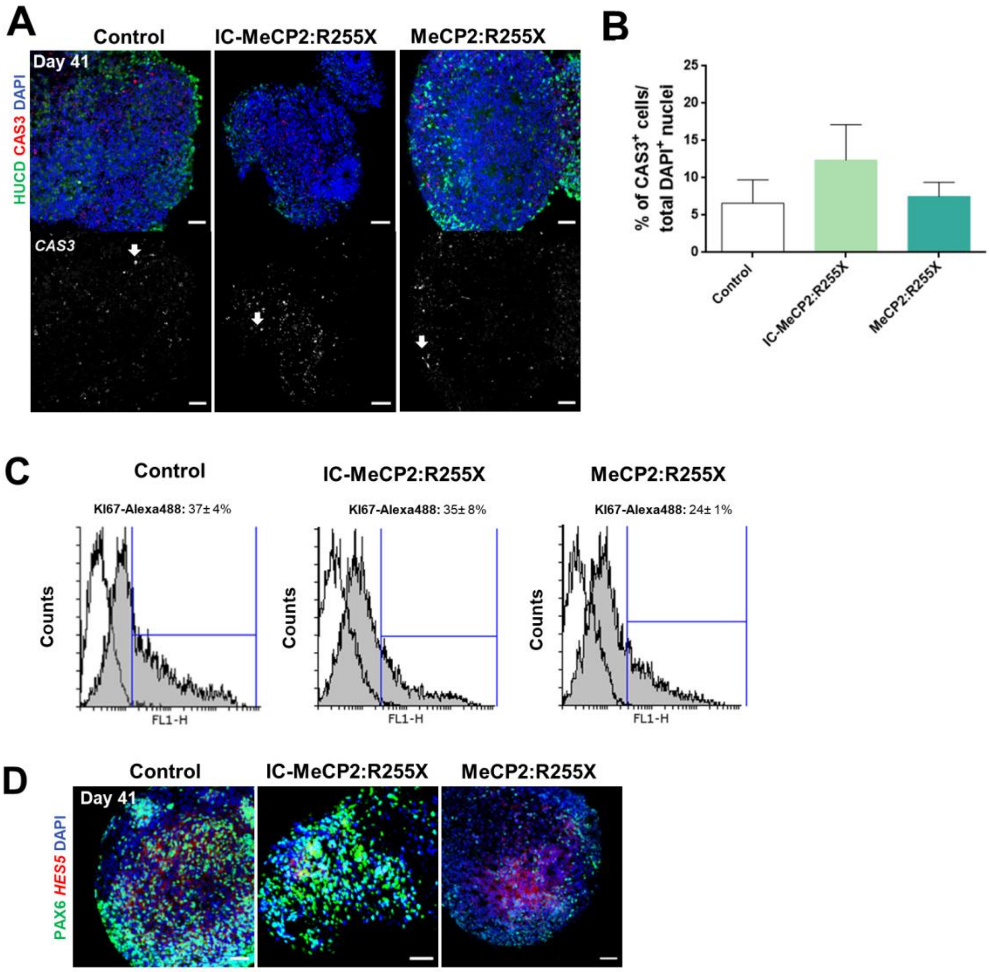

Supplementary Figure 2. Cell apoptosis and altered Notch signaling dynamics during differentiation of RTT female patient-derived dorsal organoids. A) Representative images of immunocytochemistry analysis for the neuronal marker HUCD and cleaved (active) CAS3, which is expressed in the apoptotic cells. **B)** CAS3 quantification from the immunofluorescence images. n=independent experiments; Control: n=10 from at least 10 distinct organoids; IC-MeCP2:R255X: n=3 from at least 6 distinct organoids; MeCP2:R255X: n=4 from at least 6 distinct organoids. Error bars represent SEM. **C)** Representative histograms of the flow cytometry analysis for Ki67. **D)** Supplementary images of *in situ* hybridization of *HES5* with co-localization with PAX6 progenitors, performed by immunofluorescence. Scale bar 50µm

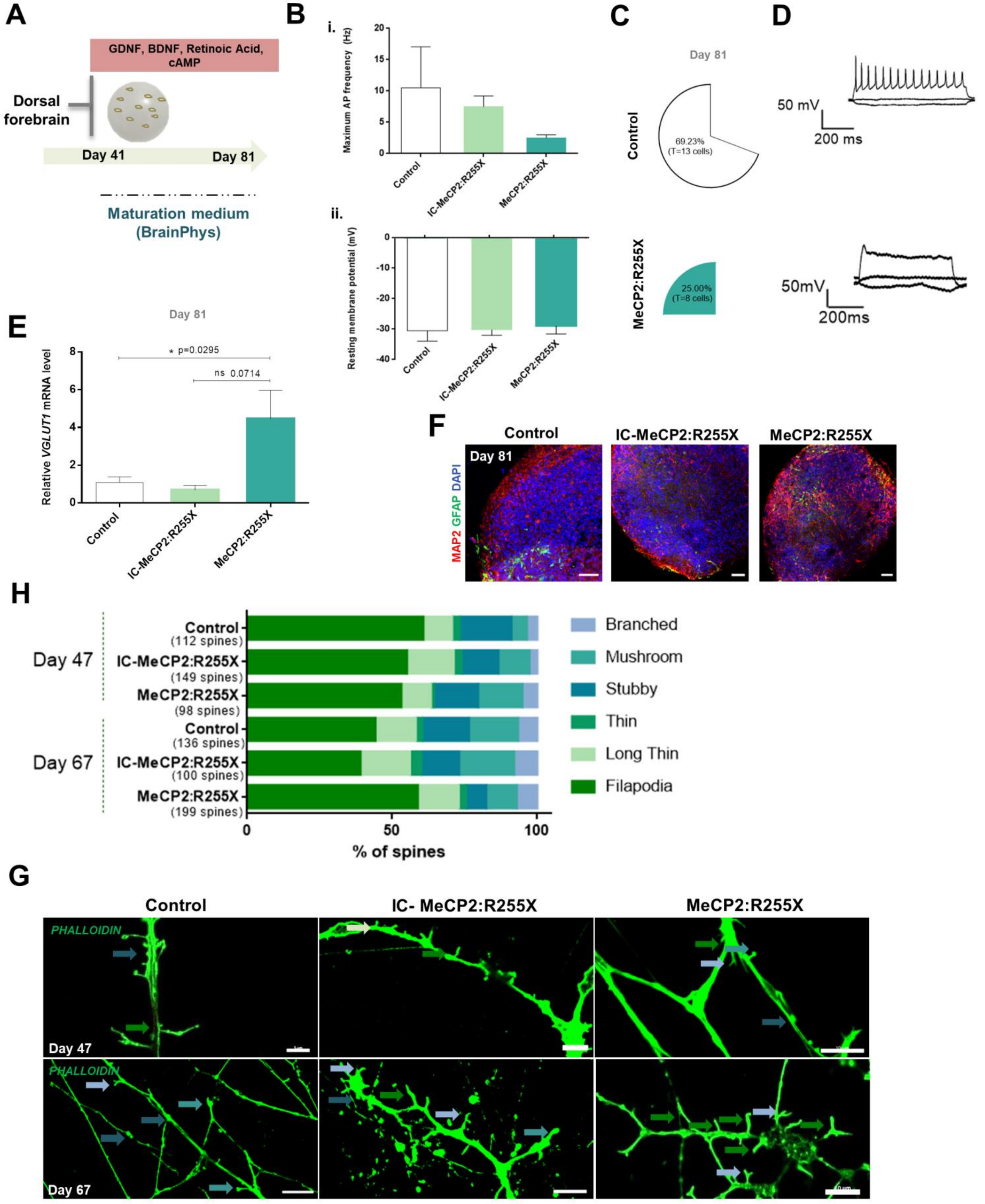
A
GDNF, BDNF, Retinoic Acid, cAMP
Dorsal forebrain
Day 41
Day 81
Maturation medium (BrainPhys)
B
i.
Maximum AP frequency (Hz)
Control
IC-MeCP2:R255X
MeCP2:R255X
ii.
Resting membrane potential (mV)
C
Day 81
Control
69.23% (T=13 cells)
MeCP2:R255X
25.00% (T=8 cells)
D
50 mV
200 ms
50mV
200ms
E
Day 81
* p=0.0295
ns 0.0714
Relative VGLUT1 mRNA level
F
Control
IC-MeCP2:R255X
MeCP2:R255X
Day 81
MAP2 GFAP DAPI
H
Day 47
Day 67
Control (112 spines)
IC-MeCP2:R255X (149 spines)
MeCP2:R255X (98 spines)
Control (136 spines)
IC-MeCP2:R255X (100 spines)
MeCP2:R255X (199 spines)
% of spines
Branched
Mushroom
Stubby
Thin
Long Thin
Filapodia
G
Control
IC- MeCP2:R255X
MeCP2:R255X
PHALLOIDIN
Day 47
Day 67

Supplementary Figure 3. Functional analysis of dorsal forebrain organoids during maturation. A) Schematic illustration of the protocol for dorsal organoid maturation until day 81. **B) i)** Firing action potential (AP) frequency and **ii)** resting membrane potential (RMP) at day 47 of differentiation. **C)** % of cells depicting at least one AP at day 81 of differentiation. The error bars represent SEM. **D)** Representative firing AP traces obtained at day 81 of firing responses evoked under current-clamp mode by injection of a 500ms current pulse (-25 to +275pA or 25-pA increments) from an initial holding potential (Vh) of -70mV. Scale bars correspond to 50pA and 200ms. **E)** qRT-PCR analysis of the glutamatergic neuronal marker *VGLUT1* (day 81). mRNA levels relative to *GAPDH* and normalized to the WT condition. n=independent experiments; Control: n=6; IC-MeCP2:R255X: n=4; MeCP2:R255X: n=4. **F)** Immunofluorescence analysis of dorsal organoid slices at day 81, for astrocyte marker GFAP and for the mature neuronal marker MAP2. Scale bar 50µm. **G)** Representative images of dendritic spine morphologies, stained with phalloidin, for each hiPSC line-derived dorsal organoid at days 47 and 67. Scale bar 5, 10 and 20µm. **H)** Relative proportion of the different dendritic spine morphologies observed at days 47 and 67 of differentiation. The total number of spines analyzed is indicated on the bar graphic. n=independent experiments; Control: n=8 from a total of 17 confocal images; IC-MeCP2:R255X: n=3 from a total of 11 confocal images; MeCP2:R255X: n=3 from a total of 17 confocal images. No statistical analysis was used.

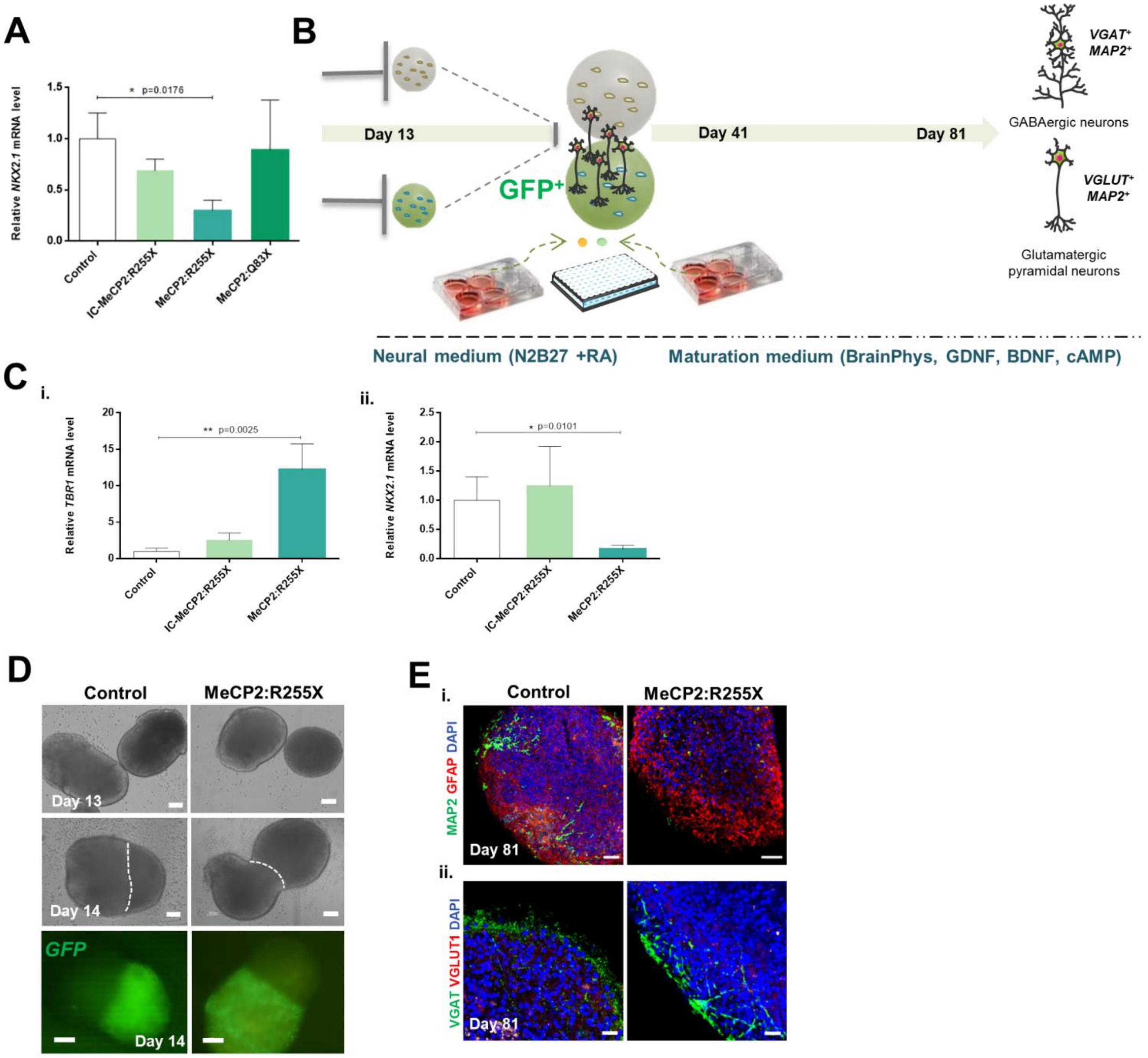

Supplementary Figure 4. Patterning of ventral forebrain organoids and fused cortical organoids characterization. A) Relative mRNA levels of *NKX2.1* represented in the heatmap of Figure 4. mRNA levels are relative to *GAPDH* and normalized to the control condition. n=independent experiments; Control: n=11; IC-MeCP2:R255X: n=4; MeCP2:R255X: n=4; MeCP2:Q83X: n=5. **B)** Schematic overview of the protocol for fusion and maintenance of the fused forebrain organoids until day 81 of culture. **C)** Relative mRNA levels of **i)** *TBR1* and **ii)** *NKX2.1* in fused forebrain organoids. mRNA levels are relative to *GAPDH* and normalized to the control condition. n=independent experiments; Control: n=11; IC-MeCP2:R255X: n=4; MeCP2:R255X: n=4; MeCP2:Q83X: n=5. **D)** Representative brightfield images of the process of fusion of ventral with dorsal organoids at day 13 and 14 of culture. Representative images of GFP^- dorsal and GFP^+ ventral organoids one day after the fusion process. Scale bar 50µm**. E) i)** Immunofluorescence staining of sections of fused organoids at day 81 for the astrocyte marker GFAP and for the neuronal

marker MAP2. **ii)** Immunofluorescence staining of organoid sections for the glutamatergic marker VGLUT1 and for the GABAergic pyramidal neuronal marker VGAT. Scale bar 50µm. For all graphics depicted, student´s t-test (two-tailed) statistics, was applied * $p<0.05$, ** $p<0.01$, *** $p<0.001$; error bars represent SEM.
